# Predicting 1-year successful clinical use of an arteriovenous access for hemodialysis using machine learning

**DOI:** 10.1038/s41746-025-02187-9

**Published:** 2025-12-04

**Authors:** Ben Li, Naomi Eisenberg, Derek Beaton, Douglas S. Lee, Leen Al-Omran, Duminda N. Wijeysundera, Mohamad A. Hussain, Ori D. Rotstein, Elisa Greco, Charles de Mestral, Muhammad Mamdani, Graham Roche-Nagle, Mohammed Al-Omran

**Affiliations:** 1 Department of Surgery, University of Toronto, Toronto, ON Canada; 2 Division of Vascular Surgery, St. Michael’s Hospital, Unity Health Toronto, Toronto, ON Canada; 3 Institute of Medical Science, University of Toronto, Toronto, ON Canada; 4 Temerty Centre for Artificial Intelligence Research and Education in Medicine (T-CAIREM), University of Toronto, Toronto, ON Canada; 5 Division of Vascular Surgery, Peter Munk Cardiac Centre, University Health Network, Toronto, ON Canada; 6 Data Science & Advanced Analytics, Unity Health Toronto, University of Toronto, Toronto, ON Canada; 7 Division of Cardiology, Peter Munk Cardiac Centre, University Health Network, Toronto, ON Canada; 8 Institute of Health Policy, Management and Evaluation, University of Toronto, Toronto, ON Canada; 9 ICES, University of Toronto, Toronto, ON Canada; 10 School of Medicine, Alfaisal University, Riyadh, Saudi Arabia; 11 Department of Anesthesia, St. Michael’s Hospital, Unity Health Toronto, Toronto, ON Canada; 12 Li Ka Shing Knowledge Institute, St. Michael’s Hospital, Unity Health Toronto, Toronto, ON Canada; 13 Division of Vascular and Endovascular Surgery and the Center for Surgery and Public Health, Brigham and Women’s Hospital, Harvard Medical School, Boston, MA USA; 14 Division of General Surgery, St. Michael’s Hospital, Unity Health Toronto, Toronto, ON Canada; 15 Leslie Dan Faculty of Pharmacy, University of Toronto, Toronto, ON Canada; 16 Division of Vascular and Interventional Radiology, University Health Network, Toronto, ON Canada; 17 Department of Surgery, King Faisal Specialist Hospital and Research Center, Riyadh, Saudi Arabia

**Keywords:** Diseases, Health care, Medical research, Nephrology

## Abstract

Arteriovenous (AV) access is important to support long-term hemodialysis; however, a significant proportion fail due to inadequate maturation or other complications. Tools that can predict long-term AV access success may guide clinical decision-making but remain limited. We developed machine learning (ML) algorithms that predict 1-year successful clinical use of an AV access using pre-operative data. The Vascular Quality Initiative (VQI) was used to identify patients who underwent surgical AV fistula/graft creation between 2011–2024. We identified 111 pre-operative demographic, clinical, and anatomic features. Six ML models were trained with 10-fold cross-validation. Overall, 59,674 patients underwent AV access creation and 28,304 (47.4%) had 1-year successful clinical use of their index AV access. The best prediction model was XGBoost, achieving an AUROC of 0.90. In comparison, logistic regression had an AUROC of 0.70. The XGBoost model accurately predicted 1-year successful clinical use of an AV access for hemodialysis, performing better than logistic regression.

## Introduction

Arteriovenous (AV) access in the form of an AV fistula or graft is important for long-term hemodialysis in patients with kidney failure requiring kidney replacement therapy^1^. In the United States, more than 800,000 individuals are on kidney replacement therapy^2^. Globally, the most common form of kidney replacement therapy is dialysis, and approximately 90% of patients who undergo dialysis are treated with hemodialysis^3^. Surgical AV access is preferred over central venous catheters (CVC) for long-term hemodialysis as it reduces the risk of infectious, thrombotic, and other complications related to a foreign body exposing the circulatory system to the environment^4^. However, up to 60% of AV accesses may not be usable for dialysis due to insufficient maturation or other complications^5^. Furthermore, the 2-year cumulative patency for AV accesses is less than 70%^5,6^. Maturation and cumulative patency rates are strongly associated with clinical decisions regarding AV access planning, including the choices of inflow artery, outflow vein, and fistula vs. graft, among others^5,6^. Furthermore, it can take weeks to months to determine whether an AV access has matured sufficiently for hemodialysis^7^. In the event of an unsuccessful AV access creation procedure, whereby the access cannot be used for hemodialysis, the patient generally requires a temporary CVC, additional access planning, and re-intervention and/or creation of a new AV access. This may lead to significant patient burden, clinical risks, and costs^8,9^. Therefore, accurate prediction of the long-term outcomes of an AV access may support better access planning, monitoring, and patient counselling to facilitate personalized care and potentially improve access outcomes.

Currently, there are no widely used tools to support the prediction of the successful clinical use of a planned AV access for long-term hemodialysis using pre-operative data. For example, Heindel et al.^10^ and Robbin et al.^11^ both used up to 6-week post-operative ultrasound measurements as input features for their models to predict AV fistula maturation and/or success rates^10,11^. Although there are good prospective data to show that post-operative ultrasound measurements can help predict AV access readiness for use, previous work has not been able to identify strong correlations between pre-operative variables and AV access outcomes, potentially due to smaller sample sizes and restrictive clinical cohorts^10,11^. These limitations could be addressed with the use of clinical registry data, such as the Vascular Quality Initiative (VQI)^12^. A pre-operative prediction model may help guide decision-making regarding the index AV access creation procedure (e.g., choices of inflow artery, outflow vein, and/or fistula vs. graft) and complement the use of existing post-operative ultrasound predictions for AV access maturation and readiness for use, supporting improved AV access creation and management. Elsewhere, the Society for Vascular Surgery (SVS) Cardiac Risk Index (CRI) predicts outcomes following aortic, carotid, and peripheral arterial interventions, but not AV access creation procedures^13^. Other tools such as the National Surgical Quality Improvement Program (NSQIP) online surgical risk calculator use modelling techniques that require manual input of clinical variables, which deters routine use in busy medical settings^14^. Therefore, there is an important need to develop better and more practical outcome prediction tools for patients being considered for AV access creation.

Machine learning (ML) is a rapidly advancing technology that allows computers to learn from data and make accurate predictions^15^. Using advanced analytics, ML can model complex relationships between inputs (e.g., patient characteristics) and outputs (e.g., clinical outcomes)^15^. This field has been driven by the explosion of electronic information combined with increasing computational capabilities^15^. The advantage of newer ML techniques over traditional statistical methods is that they can better model complex, multicollinear relationships between covariates and outcomes^16^, which is common in health care data^17^. Previously, ML was applied to the NSQIP database to develop an algorithm that predicts peri-operative outcomes for over 2,900 distinct procedures^18^. Given the heterogeneity of this cohort, better predictive performance may be achieved by building ML algorithms specific to patients undergoing AV access creation using the VQI database, a dedicated vascular registry containing procedure-specific variables^12^. We previously described ML algorithms trained on VQI data for predicting outcomes following aortic, carotid, and peripheral arterial interventions, which achieved better performance compared to traditional statistical techniques such logistic regression and existing tools^19–21^. The development of a ML-based prediction algorithm for AV access outcomes may complement these existing models and expand clinical guidance for the management of patients with end stage kidney disease (ESKD). In this study, we used VQI data to develop ML algorithms that predict 1-year outcomes following AV access creation using pre-operative data. Secondarily, we performed sensitivity analyses by training additional models using intra- and post-operative variables to determine their relative impact on model performance compared to using pre-operative data alone. We hypothesized that our ML models could achieve good predictive performance using only pre-operative data and achieve better performance than traditional statistical techniques such as logistic regression.

## Results

### Patients, events, and follow-up

From an initial cohort of 60,975 patients who underwent AV access creation for hemodialysis in the VQI database between August 1, 2011 and January 2, 2024, a total of 1,301 patients were excluded for the following reasons: no reported access type of fistula or graft (*n* = 123), inflow artery (*n* = 762), or outflow vein (*n* = 367), or the patient underwent endovascular AV fistula creation (*n* = 49). The final analysis included 59,674 patients who underwent AV access creation, including 45,571 (76.4%) fistulae and 14,101 (23.6%) grafts. Most AV accesses were created in the upper arm at or above the elbow (n = 29,256, 49.0%), followed by the forearm below the elbow (*n* = 27,852, 46.7%), lower extremity (*n* = 1,020, 1.7%), or other anatomic location (*n* = 1,546, 2.6%). Overall, 28,304 (47.4%) had 1-year successful clinical use of the AV access for hemodialysis. For secondary outcomes, AV access thrombosis occurred in 3,553 (6.0%) patients and AV access re-interventions occurred in 9,684 (16.2%) patients over 1 year of follow-up. For re-interventions, 7429 (12.4%) patients had surgical re-interventions, and 4389 (7.4%) patients had endovascular re-interventions. Mean follow-up was 15.7 (SD 1.9) months.

### Pre-operative characteristics

Compared to patients without a primary outcome, those who had 1-year successful clinical use of the AV access for hemodialysis were younger (mean age 61.2 [SD 14.7] vs. 62.6 [SD 14.6] years, *p* < 0.001), less likely to be female (41.9% vs. 45.4%, *p* < 0.001), had a lower BMI, and were more likely to be Asian, Black, or Hispanic. They were also more likely to receive Medicaid and have their AV access created in an ambulatory center rather than a hospital and less likely to be transferred from another hospital or from a rehabilitation unit to the center of intervention. Patients who had 1-year successful clinical use of the AV access were less likely to be prior or current smokers or have diabetes, coronary artery disease (CAD), congestive heart failure (CHF), dysrhythmia, chronic obstructive pulmonary disease (COPD), and an American Society of Anesthesiologists (ASA) class ≥ 4. They were also more likely to have chronic kidney disease (CKD) stage 5 and be on hemodialysis rather than at the pre-dialysis stage. Functionally, patients with 1-year successful clinical use of the AV access were more likely to live at home and ambulate independently. For laboratory investigations, they had a higher mean creatinine and lower estimated glomerular filtration rate (eGFR). In terms of medications, they were less likely to be on acetylsalicylic acid, P2Y12 antagonists, and statins, and more likely to be on anticoagulants. For vascular access history, patients with 1-year successful clinical use of the AV access were more likely to have had prior AV fistulae or grafts in any anatomic location. They were also more likely to receive pre-operative imaging, including arterial duplex, arteriogram, ultrasound vein mapping, and venogram for vascular access planning. Patients with 1-year successful clinical use of the AV access had higher mean pre-operative diameters for their target inflow artery and outflow vein based on imaging. They were also more likely to receive an AV fistula rather than a graft and an upper arm or lower extremity access rather than a forearm access (Table 1).

**Table 1:** Pre-operative demographic and clinical characteristics of patients undergoing arteriovenous access creation with and without 1-year successful clinical use of the index access for hemodialysis

	Index access not used for hemodialysis (abandoned) at 1-year of follow-up (*n* = 31,370)	Successful clinical use of the index access for hemodialysis at 1-year of follow-up (*n* = 28,304)	*P*
**Demographics**			
Age, years, mean (SD)	62.6 (14.6)	61.2 (14.7)	< 0.001
Female	14,228 (45.4)	11,853 (41.9)	< 0.001
BMI, kg/m^2^, mean (SD)	30.4 (8.4)	29.7 (7.8)	< 0.001
Race			
American Indian or Alaskan Native	383 (1.2)	416 (1.5)	< 0.001
Asian	871 (2.8)	1,078 (3.8)	
Black	10,319 (32.9)	10,379 (36.7)	
Native Hawaiian or other Pacific Islander	136 (0.4)	130 (0.5)	
White	17,329 (55.2)	13,996 (49.4)	
More than 1 race	173 (0.6)	175 (0.6)	
Unknown/other	2159 (6.9)	2089 (7.4)	
Hispanic ethnicity	2595 (8.3)	2952 (10.4)	< 0.001
Insurance status			
Medicare	17,080 (54.5)	15,052 (53.2)	< 0.001
Medicaid	3592 (11.5)	3500 (12.4)	
Commercial	8678 (27.7)	7993 (28.2)	
Military/Veterans Affairs	272 (0.9)	192 (0.7)	
Non-US Insurance	507 (1.6)	378 (1.3)	
Self-pay (uninsured)	589 (1.9)	610 (2.2)	
Unknown/other	652 (2.1)	579 (2.1)	
Rural residence	881 (2.8)	728 (2.6)	0.08
Area Deprivation Index percentile, median (IQR)	58 (36 – 78)	58 (33 – 80)	0.15
Performance site			
Hospital outpatient	24,784 (79.0)	22,147 (78.2)	< 0.001
Hospital inpatient	5457 (17.4)	4614 (16.3)	
Ambulatory center	1093 (3.5)	1491 (5.3)	
Office	32 (0.1)	47 (0.2)	
Not reported	4 (0.01)	5 (0.02)	
Transfer status			
From another hospital	498 (1.6)	307 (1.1)	< 0.001
From rehabilitation unit	190 (0.6)	121 (0.4)	
**Comorbidities**			
Smoking status			
Never	15,067 (48.0)	14,173 (50.1)	< 0.001
Prior	11,746 (37.4)	10,070 (35.6)	
Current	4,557 (14.5)	4,061 (14.3)	
Hypertension	29,382 (93.7)	26,637 (94.1)	< 0.001
Diabetes	19,141 (61.0)	17,127 (60.5)	< 0.001
Coronary artery disease	8091 (25.8)	6575 (23.2)	< 0.001
Prior coronary artery bypass	3234 (10.3)	2594 (9.2)	< 0.001
Prior percutaneous coronary intervention	4084 (13.0)	3310 (11.7)	< 0.001
Congestive heart failure	9947 (31.7)	8543 (30.2)	< 0.001
Dysrhythmia	2349 (7.5)	1458 (5.2)	< 0.001
Chronic obstructive pulmonary disease			
Not treated	1134 (3.6)	1013 (3.6)	< 0.001
On medications	3780 (12.0)	2825 (10.0)	
On home oxygen	1305 (4.2)	851 (3.0)	
Peripheral artery disease	24,148 (77.0)	22,957 (81.1)	< 0.001
Intravenous drug use			
Past	474 (1.5)	425 (1.5)	0.001
Current	197 (0.6)	116 (0.4)	
HIV status			
HIV+	287 (0.9)	289 (1.0)	< 0.001
HIV+ with current infection	53 (0.2)	78 (0.3)	
CKD stage			
1	4 (0.01)	5 (0.02)	< 0.001
2	4 (0.01)	8 (0.03)	
3	118 (0.4)	63 (0.2)	
4	2266 (7.2)	1259 (4.5)	
5	28,978 (92.4)	26,969 (95.3)	
Dialysis status			
Pre-dialysis	11,648 (37.1)	7548 (26.7)	< 0.001
On hemodialysis	19,347 (61.7)	20,485 (72.4)	
On peritoneal dialysis	176 (0.6)	117 (0.4)	
Functioning transplant, never on dialysis	133 (0.4)	110 (0.4)	
Functioning transplant, previously on dialysis	56 (0.2)	29 (0.1)	
Not reported	10 (0.03)	15 (0.05)	
ASA class			
1	77 (0.2)	44 (0.2)	< 0.001
2	888 (2.8)	819 (2.9)	
3	18,295 (58.3)	16,994 (60.0)	
4	11,976 (38.2)	10,346 (36.6)	
5	26 (0.08)	16 (0.06)	
Not reported	108 (0.3)	85 (0.3)	
**Functional status**			
Living status			
Home	29,852 (95.2)	27,267 (96.3)	< 0.001
Nursing home	1385 (4.4)	926 (3.3)	
Homeless	86 (0.3)	80 (0.3)	
Not reported	47 (0.2)	31 (0.1)	
Ambulatory status			
Ambulatory independently	26,174 (83.4)	23,765 (84.0)	< 0.001
Ambulatory with assistance	3541 (11.3)	3204 (11.3)	
Wheelchair-dependent	1458 (4.7)	1208 (4.3)	
Bedridden	197 (0.6)	127 (0.4)	
**Laboratory investigations**			
Hemoglobin, g/L, mean (SD)	107.0 (44.8)	106.0 (17.5)	< 0.001
Creatinine, umol/L, mean (SD)	408.0 (98.3)	427.0 (93.4)	< 0.001
Estimated glomerular filtration rate, mL/min/1.73 m^2^, mean (SD)	13.1 (1.9)	12.9 (1.5)	< 0.001
**Medications**			
Acetylsalicylic acid	4385 (14.0)	3417 (12.1)	< 0.001
P2Y12 antagonist	1145 (3.6)	902 (3.2)	0.005
Statin	6389 (20.4)	4687 (16.6)	< 0.001
Anticoagulant	777 (2.5)	1233 (4.4)	< 0.001
**Vascular access history**			
Number of prior AV fistula creation procedures			
0	27,782 (88.6)	24,381 (86.1)	< 0.001
1	2704 (8.6)	2980 (10.5)	
2	675 (2.2)	727 (2.6)	
3	158 (0.5)	157 (0.6)	
4 or more	51 (0.2)	59 (0.2)	
Number of prior AV graft creation procedures			
0	29,919 (95.4)	26,761 (94.5)	< 0.001
1	1155 (3.7)	1193 (4.2)	
2	233 (0.7)	284 (1.0)	
3	39 (0.1)	55 (0.2)	
4 or more	24 (0.08)	11 (0.04)	
Previous forearm fistula	2835 (9.0)	3037 (10.7)	< 0.001
Previous upper arm fistula	3093 (9.9)	3313 (11.7)	< 0.001
Previous forearm graft	731 (2.3)	854 (3.0)	< 0.001
Previous upper arm graft	1374 (4.4)	1612 (5.7)	< 0.001
Previous lower extremity AV access	318 (1.0)	360 (1.3)	0.03
Other AV access	1602 (5.1)	1573 (5.6)	0.01
**Imaging and anatomic characteristics**			
Pre-operative imaging			
Arterial duplex	10,734 (34.2)	10,408 (36.8)	< 0.001
Arteriogram	428 (1.4)	460 (1.6)	0.009
Ultrasound vein mapping	26,836 (85.5)	24,354 (86.0)	0.08
Venogram	2289 (7.3)	3037 (10.7)	< 0.001
Pre-operative target inflow artery diameter, mm, mean (SD)	4.09 (0.41)	4.21 (0.38)	< 0.001
Pre-operative target outflow vein diameter, mm, mean (SD)	3.59 (0.84)	3.74 (0.92)	< 0.001
Access type			
AV fistula	24,668 (78.6)	22,903 (80.9)	< 0.001
AV graft	6700 (21.4)	5401 (19.1)	
Inflow artery			
Radial	8134 (25.9)	6378 (22.5)	< 0.001
Ulnar	80 (0.3)	56 (0.2)	
Brachial	21,830 (69.6)	20,361 (71.9)	
Axillary	596 (1.9)	669 (2.4)	
Common femoral	232 (0.7)	260 (0.9)	
Superficial femoral	229 (0.7)	293 (1.0)	
Other	269 (0.9)	287 (1.0)	
Outflow vein			
Cephalic, forearm	13,789 (44.0)	11,819 (41.8)	< 0.001
Cephalic, upper arm	3711 (11.5)	3216 (11.4)	
Basilic, forearm	1309 (4.2)	935 (3.3)	
Basilic, upper arm	6245 (19.9)	5768 (20.4)	
Brachial	1346 (4.3)	1339 (4.7)	
Axillary	3717 (11.8)	3914 (13.8)	
Saphenous	96 (0.3)	115 (0.4)	
Femoral	374 (1.2)	435 (1.5)	
Other	783 (2.5)	763 (2.7)	
Pre-operative endovascular arterial intervention (angioplasty/stent) to treat inflow stenosis	193 (0.6)	205 (0.7)	0.11
Pre-operative endovascular venous intervention (angioplasty/stent) to treat outflow stenosis	418 (1.3)	436 (1.5)	0.04

Values are reported as No. (%) unless otherwise indicated. *ASA* (American Society of Anesthesiologists), *AV* (arteriovenous), *BMI* (body mass index), *CKD* (chronic kidney disease), *HIV* (human immunodeficiency virus), *IQR* (interquartile range), *SD* (standard deviation).

### Intra-operative characteristics

Patients with 1-year successful clinical use of the AV access were more likely to receive regional anesthesia and have higher mean inflow artery and outflow vein diameters measured intraoperatively. For concomitant procedures, patients with the primary outcome were more likely to receive a patch angioplasty on the target artery and/or vein. They were also more likely to have a completion doppler or fistulogram study intra-operatively to confirm technical success (not a part of pre-operative imaging) (Table 2).

**Table 2:** Intra-operative characteristics of patients undergoing arteriovenous access creation with and without 1-year successful clinical use of the index access for hemodialysis

	Index access not used for hemodialysis (abandoned) at 1-year of follow-up (*n* = 31,370)	Successful clinical use of the index access for hemodialysis at 1-year of follow-up (*n* = 28,304)	P
Anesthesia			
Local	9326 (29.7)	7835 (27.7)	< 0.001
Regional	9447 (30.1)	9257 (32.7)	
General	12,540 (40.0)	11,149 (39.4)	
Not reported	57 (0.2)	63 (0.2)	
Antibiotics given within 1 hour of intervention	25,714 (82.0)	23,200 (82.0)	0.75
Antibiotics stopped within 24 h of intervention	25,325 (80.7)	22,950 (81.1)	0.02
Intra-operative inflow artery diameter, mm, mean (SD)	2.78 (0.50)	2.95 (0.34)	< 0.001
Intra-operative outflow vein diameter, mm, mean (SD)	3.04 (0.51)	3.25 (0.35)	< 0.001
Concomitant procedures			
Venous angioplasty	50 (0.2)	39 (0.1)	0.56
Venous stent	17 (0.05)	21 (0.07)	0.42
Arterial angioplasty	8 (0.03)	6 (0.02)	0.94
Arterial stent	1 (0.003)	4 (0.01)	0.31
Arterial endarterectomy	22 (0.07)	9 (0.03)	0.06
Venous branch ligation	539 (1.7)	448 (1.6)	0.21
Surgical access patch angioplasty	20 (0.06)	4 (0.01)	0.005
Superficialization	152 (0.5)	134 (0.5)	0.89
Lipectomy	3 (0.01)	3 (0.01)	0.99
Other	516 (1.6)	382 (1.4)	0.003
Completion study			
Doppler	5,206 (16.6)	4,984 (17.6)	0.001
Duplex ultrasound	85 (0.3)	97 (0.3)	0.13
Fistulogram	475 (1.5)	507 (1.8)	0.009

Values are reported as No. (%) unless otherwise indicated.

*SD* (standard deviation).

### Post-operative characteristics

Immediate post-operative complications not requiring access ligation or abandonment, including ischemic steal, access thrombosis, or other complications requiring re-intervention, were rare (~2%) and occurred less frequently in patients with 1-year successful clinical use of the AV access. Mean hospital length of stay was lower in patients with the primary outcome. These patients were less likely to be discharged on acetylsalicylic acid, P2Y12 antagonists, statins, and anticoagulants. Patients with 1-year successful clinical use of the AV access were less likely to have a non-home discharge (Table 3).

**Table 3:** Post-operative in-hospital characteristics and complications of patients undergoing arteriovenous access creation with and without 1-year successful clinical use of the index access for hemodialysis

	Index access not used for hemodialysis (abandoned) at 1-year of follow-up (*n* = 31,370)	Successful clinical use of the index access for hemodialysis at 1-year of follow-up (*n* = 28,304)	P
Immediate post-operative complications not requiring access ligation or abandonment	655 (2.1)	477 (1.9)	< 0.001
Bleeding	565 (1.8)	453 (1.7)	0.059
Ischemic steal	35 (0.1)	6 (0.02)	< 0.001
Access thrombosis	32 (0.1)	8 (0.03)	< 0.001
Other complication requiring re-intervention	23 (0.07)	10 (0.04)	0.07
Bleeding management			
Resolved spontaneously	386 (1.2)	301 (1.2)	0.23
Resolved with medical treatment	46 (0.1)	35 (0.1)	
Required surgical or endovascular re-intervention	133 (0.5)	117 (0.4)	
Ischemic steal management			
Observation	5 (0.02)	0	< 0.001
Banding	2 (0.006)	1 (0.004)	
Proximalization	19 (0.06)	2 (0.007)	
Revascularization using distal inflow	2 (0.006)	1 (0.004)	
Distal revascularization and interval ligation	3 (0.01)	1 (0.004)	
Distal revascularization	4 (0.01)	1 (0.004)	
Thrombosis management			
Open surgical thrombectomy	9 (0.03)	4 (0.01)	0.35
Endovascular mechanical thrombectomy	4 (0.01)	2 (0.007)	0.78
Thrombolysis	9 (0.03)	1 (0.004)	0.02
Surgical revision	10 (0.03)	2 (0.007)	0.02
Hospital length of stay, days, mean (SD)	2.59 (1.60)	1.98 (2.53)	< 0.001
Discharge medications			
Acetylsalicylic acid	14,494 (46.2)	12,904 (45.6)	< 0.001
P2Y12 antagonist	3878 (12.4)	3377 (11.9)	< 0.001
Statin	6468 (20.6)	4764 (16.8)	< 0.001
Anticoagulant	4995 (15.9)	3742 (13.2)	< 0.001
Non-home discharge	1692 (5.4)	1138 (4.0)	< 0.001

Values are reported as No. (%) unless otherwise indicated.

*SD* (standard deviation).

### **Model performance**

Of the 6 ML models evaluated on test set data with only pre-operative variables as input features, Extreme Gradient Boosting (XGBoost) had the best performance in predicting 1-year successful clinical use of the index AV access for hemodialysis based on the primary model evaluation metric of area under the receiver operating characteristic curve (AUROC), achieving an AUROC of 0.90 [95% CI 0.89–0.91]. In comparison, the other models had the following AUROC’s [95% CI’s]: random forest (0.88 [0.87–0.89]), radial basis function support vector machine (RBF SVM) (0.85 [0.84–0.86]), Naïve Bayes (0.84 [0.83–0.85]), multilayer perceptron artificial neural network (MLP ANN) (0.77 [0.76–0.78]), and logistic regression (0.70 [0.69–0.71)]. The secondary performance metrics of XGBoost were the following: accuracy 0.82 (95% CI 0.81–0.83), sensitivity 0.82, specificity 0.81, positive predictive value (PPV) 0.81, and negative predictive value (NPV) 0.82. Model performance results are summarized in Table 4.

**Table 4:** Model performance on test set data for predicting 1-year successful clinical use of an arteriovenous access for hemodialysis using pre-operative features

	AUROC (95% CI)	Accuracy (95% CI)	Sensitivity	Specificity	PPV	NPV
XGBoost	0.90 (0.89 – 0.91)	0.82 (0.81 – 0.83)	0.82	0.81	0.81	0.82
Random forest	0.88 (0.87 – 0.89)	0.80 (0.79 – 0.81)	0.80	0.80	0.81	0.80
RBF SVM	0.85 (0.84 – 0.86)	0.77 (0.76 – 0.78)	0.79	0.75	0.73	0.80
Naïve Bayes	0.84 (0.83 – 0.85)	0.76 (0.75 – 0.77)	0.77	0.75	0.75	0.77
MLP ANN	0.77 (0.76 – 0.78)	0.70 (0.69 – 0.71)	0.72	0.68	0.67	0.74
Logistic regression	0.70 (0.69 – 0.71)	0.62 (0.61 – 0.63)	0.61	0.71	0.55	0.57

*XGBoost* (Extreme Gradient Boosting), *AUROC* (area under the receiver operating characteristic curve), *CI* (confidence interval), *PPV* (positive predictive value), *NPV* (negative predictive value), *RBF* *SVM* (radial basis function support vector machine), *MLP ANN* (multilayer perceptron artificial neural network).

**Fig. 1:**
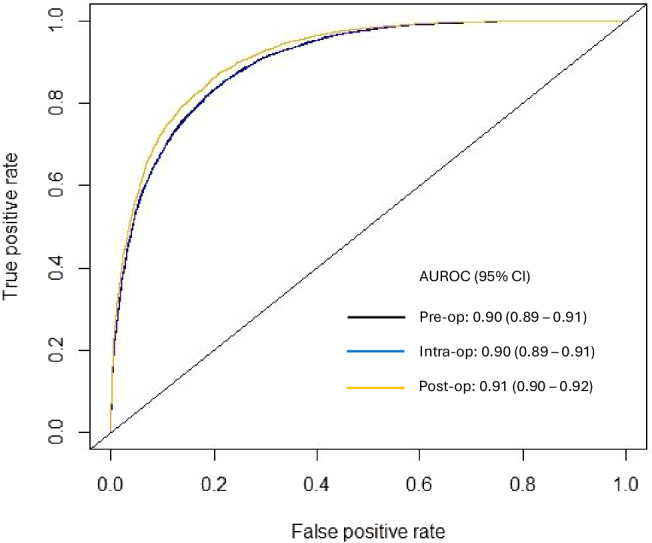
Receiver operating characteristic curve for predicting 1-year successful clinical use of an arteriovenous access for hemodialysis using Extreme Gradient Boosting (XGBoost) models at the pre-, intra-, and post-operative stages. AUROC (area under the receiver operating characteristic curve), CI (confidence interval).

**Fig. 2:**
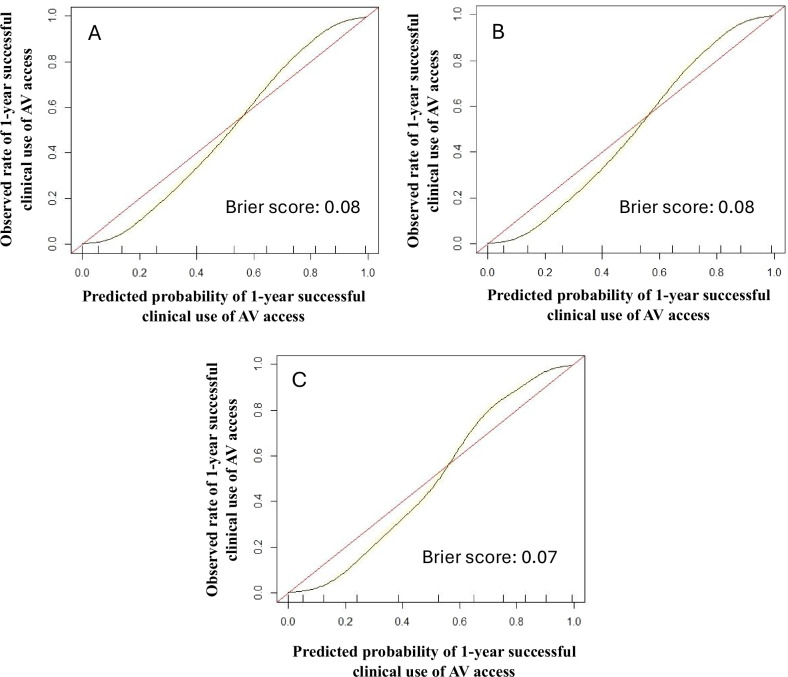
Calibration plots with Brier scores for predicting 1-year successful clinical use of an arteriovenous access for hemodialysis using Extreme Gradient Boosting (XGBoost) models at the A) pre-operative, B) intra-operative, and C) post-operative stages. Abbreviation: AV (arteriovenous).

**Fig. 3:**
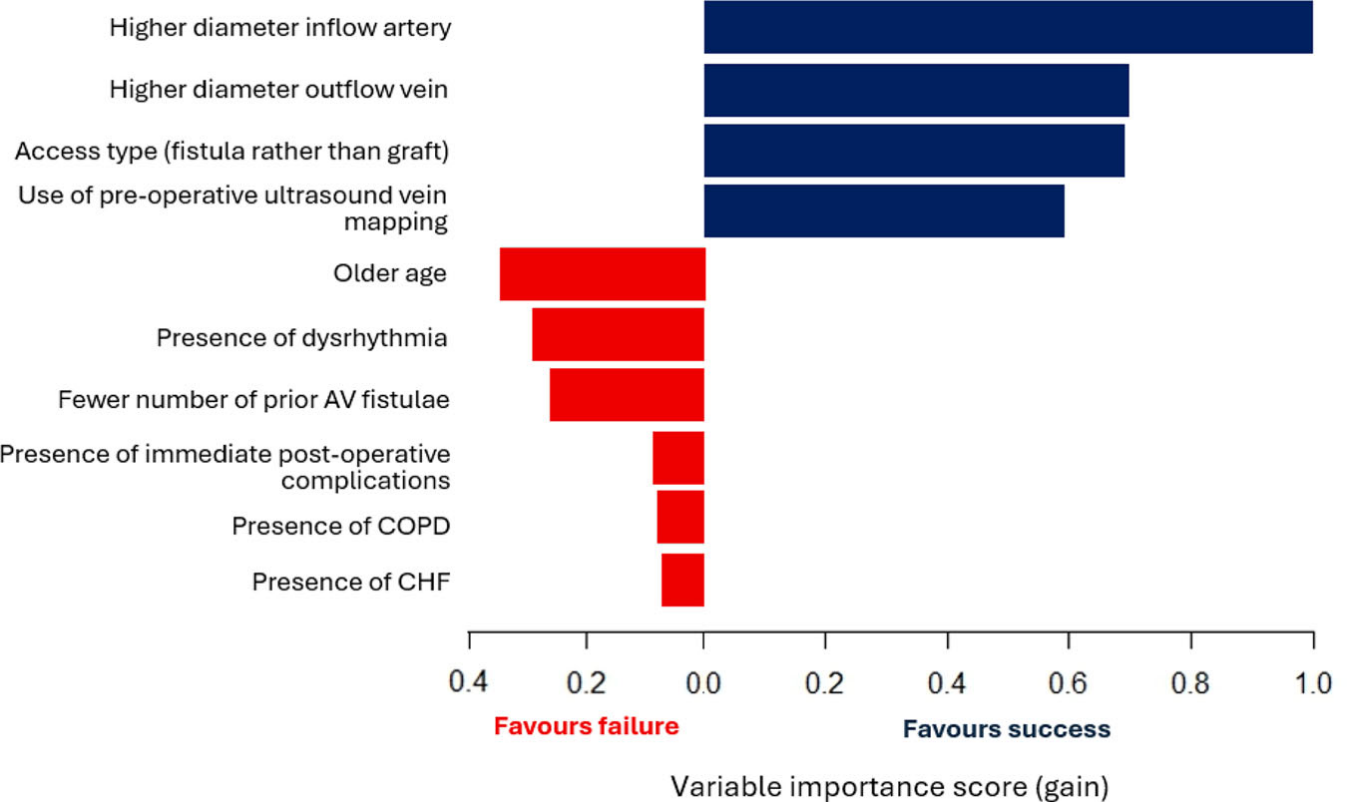
Variable importance scores (gain) for the top 10 predictors of 1-year successful clinical use of an arteriovenous access for hemodialysis in the Extreme Gradient Boosting (XGBoost) model at the post-operative stage. Abbreviations: AV (arteriovenous), COPD (chronic obstructive pulmonary disease), CHF (congestive heart failure).

For sensitivity analyses, we trained additional XGBoost models using intra- and post-operative data. The addition of intra-operative features to pre-operative features did not change model performance, with the AUROC remaining at 0.90 (95% CI 0.89–0.91), *p* = 0.90. Adding post-operative features marginally improved model performance without reaching statistical significance (AUROC 0.91, 95% CI 0.90–0.92, *p* = 0.41). The ROC curves are presented in Fig. 1, demonstrating excellent predictive performance of the XGBoost model for 1-year successful clinical use of an index AV access for hemodialysis at the pre-, intra-, and post-operative stages. For secondary outcomes, the XGBoost model predicted 1-year AV access thrombosis and re-interventions with AUROC’s of 0.86 (95% CI 0.85–0.87) and 0.88 (95% CI 0.87–0.89) at both the pre- and intra-operative stages, respectively (Table 5). The XGBoost model was not trained to predict the secondary outcomes using post-operative variables because some post-operative variables were directly related to thrombosis and/or re-intervention and would therefore artificially elevate model performance. Given that excellent model discrimination can sometimes be accompanied by poor calibration, we also assessed the calibration of our models. There was good agreement between predicted and observed event probabilities as demonstrated by the calibration plots in Fig. 2A–C, with Brier scores of 0.08 (pre-operative), 0.08 (intra-operative), and 0.07 (post-operative). This highlights the excellent calibration of the XGBoost model across the entire probability range for predicting 1-year successful clinical use of an index AV access for hemodialysis at the pre-, intra-, and post-operative stages. The top 10 predictors of 1-year successful clinical use of the index AV access for hemodialysis in the final XGBoost model included 9 pre-operative features (planned inflow artery and outflow vein, planned access type, pre-operative ultrasound vein mapping, age, dysrhythmia, number of prior AV fistulae, COPD, and CHF), and 1 post-operative feature (immediate post-operative complications). Four features predicted AV access success: higher diameter inflow artery, higher diameter inflow vein, access type of fistula rather than graft, and use of pre-operative ultrasound vein mapping. In contrast, six features predicted AV access failure: older age, presence of dysrhythmia, fewer number of prior AV fistulae, presence of immediate post-operative complications, presence of COPD, and presence of CHF (Fig. 3). These results highlight that anatomic features (inflow artery and outflow vein diameters), pre-operative planning (decision on fistula vs. graft and use of pre-operative imaging), and patient history (comorbidities and vascular access history) are critical in determining long-term AV access success.

**Table 5:** XGBoost performance on test set data for predicting 1-year primary and secondary outcomes following arteriovenous access creation at the pre-, intra-, and post-operative stages

	AUROC (95% CI)	Accuracy (95% CI)	Sensitivity	Specificity	PPV	NPV
**Successful clinical use of the index AV access for hemodialysis (primary outcome)**						
Pre-op	0.90 (0.89 – 0.91)	0.82 (0.81 – 0.83)	0.82	0.81	0.81	0.82
Intra-op	0.90 (0.89 – 0.91)	0.82 (0.81 – 0.83)	0.83	0.81	0.81	0.82
Post-op	0.91 (0.90 – 0.92)	0.83 (0.82 – 0.84)	0.85	0.81	0.80	0.86
**AV access thrombosis**						
Pre-op	0.86 (0.85 – 0.87)	0.80 (0.79 – 0.81)	0.80	0.80	0.79	0.82
Intra-op	0.86 (0.85 – 0.87)	0.80 (0.79 – 0.81)	0.80	0.80	0.80	0.82
Post-op*	-	-	-	-	-	-
**Re-intervention on AV access**						
Pre-op	0.88 (0.87 – 0.89)	0.81 (0.80 – 0.82)	0.82	0.75	0.78	0.85
Intra-op	0.88 (0.87 – 0.89)	0.81 (0.80 – 0.82)	0.82	0.77	0.78	0.85
Post-op*	-	-	-	-	-	-

^*^Models were not trained to predict AV access thrombosis or re-intervention using post-operative variables because some post-operative variables were directly related to thrombosis and/or re-intervention, which would artificially elevate model performance.

*AV* (arteriovenous), *XGBoost* (Extreme Gradient Boosting), *AUROC* (area under the receiver operating characteristic curve), *CI* (confidence interval), *PPV* (positive predictive value), *NPV* (negative predictive value), *pre-op* (pre-operative), *intra-op* (intra-operative), *post-op* (post-operative).

### Subgroup analysis

Model performance remained robust on subgroup analyses across demographic/clinical populations based on age, sex, race, ethnicity, rurality, median area deprivation index (ADI) percentile, access type (fistula or graft), and access location (forearm, upper arm, lower extremity, or other), with AUROC’s ranging from 0.89-0.91 and no significant differences between majority and minority groups (Supplementary Figs. 1–8).

## Discussion

We used data from a large clinical registry (VQI) consisting of 59,674 patients who underwent surgical AV fistula or graft creation to develop ML models that can accurately predict 1-year successful clinical use of the index AV access for hemodialysis with AUROC’s ≥0.90 using pre-operative data. There were several key findings. First, patients with successful AV accesses have predictive features at the pre-, intra-, and post-operative stages. ML-based modeling allowed us to determine the relative impact of these factors on AV access success. Second, we evaluated 6 ML models on our dataset and XGBoost achieved the best performance, demonstrating excellent discrimination and calibration. Furthermore, predictive performance remained robust across demographic and clinical subpopulations. Third, while intra- and post-operative factors contributed to long-term risk, 9 of the top 10 predictors for 1-year successful clinical use of the AV access for hemodialysis were pre-operative features. Additionally, the addition of intra-operative features to the pre-operative model did not change predictive performance and the addition of post-operative features marginally improved model performance. This highlights an important opportunity for our risk prediction tool to guide pre-operative AV access planning and procedural selection. Overall, these models have the potential to support clinical decision-making both pre-operatively and throughout a patient’s perioperative course, thereby facilitating individualized risk assessment, counselling, monitoring, and management.

Heindel and colleagues^10^ used pooled patient-level data from 704 patients undergoing new radiocephalic AV fistula creation from the PATENCY-1 and PATENCY-2 randomized controlled trials to develop ML models that predict 1-year successful unassisted AV fistula use with AUROC’s ranging from 0.78–0.81 and accuracies ranging from 69.1–73.6%^10^. They demonstrated that the performance of their models was superior to the National Kidney Foundation Kidney Disease Outcomes Quality Initiative (KDOQI) and University of Alabama at Birmingham ultrasound threshold criteria^10^. There were several key differences between our studies. First, while Heindel and colleagues^10^ used randomized controlled trial data consisting of 704 patients, we used clinical registry data consisting of 59,674 patients^10^. While trial data have several advantages including prospective follow-up, adjudicated AV access outcomes, rigorous data collection methods, low missingness, high internal validity, and low misclassification of predictors and outcomes, registry data contains larger sample sizes critical for robust ML model training and evaluation and may better reflect real-world outcomes^10^. Second, Heindel and colleagues^10^ developed prediction models for radiocephalic AV fistulae, while we developed prediction models for all types of upper and lower extremity AV fistulae and grafts^10^. Models built for specific access configurations benefit from more granular data but may be less generalizable^10^. Although our approach may increase heterogeneity, it increases applicability of our model for AV access planning, as clinicians must consider different AV access approaches to determine the optimal strategy. Importantly, we demonstrated through subgroup analyses that our models performed well when evaluated specifically on AV fistulae vs. grafts and based on anatomic location (forearm vs. upper arm vs. lower extremity vs. other). Third, while we developed intra- and post-operative prediction models, our focus was on the ability for our models to make accurate predictions regarding long-term AV access success using pre-operative variables alone. In contrast, Heindel and colleagues^10^ used 4–6 week post-operative ultrasound parameters in addition to baseline characteristics as model input features^10^. While post-operative ultrasound parameters have important associations with long-term AV access success, a pre-operative prediction model may additionally support clinical decision-making and procedural planning for the index access creation intervention. In our study, we demonstrated that the use of pre-operative demographic and clinical variables alone from a large clinical registry allowed us to build highly accurate predictive ML models with an AUROC of 0.90 and accuracy of 82% for 1-year AV access success. Fourth, while Heindel and colleagues^10^ predicted the primary outcome of 1-year successful unassisted AV fistula use^10^, our models were trained to predict 1-year successful clinical use of the index AV access for hemodialysis, with or without re-interventions. While unassisted AV fistula use is an important outcome, it is well-established that AV accesses may require relatively low-risk re-interventions to support maturation and/or maintain patency^22^. Therefore, there is important clinical value of AV accesses that remain functional with re-interventions. Additionally, our models were trained to predict the secondary outcomes of re-intervention and thrombosis, which are of clinical importance to vascular surgeons, nephrologists, and other clinicians. Overall, we build on the work of Heindel and colleagues^10^ by using registry data with larger sample sizes, allowing us to develop pre-operative prediction models that can be used in conjunction with post-operative ultrasound measurements to optimize AV access success rates^10^.

Our findings regarding the important predictors of long-term AV access success align with the existing literature^1^. Fedorova and colleagues^23^ analyzed VQI data from 2011-2019 and found a significant association between pre-operative vein mapping and the incidence of the loss of secondary patency at 1 year of follow-up^23^. Similarly, we demonstrated that pre-operative vein mapping was one of the most important input features in our model for predicting 1-year successful clinical use of the AV access for hemodialysis. Like Heindel and colleagues^10^, who demonstrated that a larger outflow vein diameter, higher flow volume, and absence of >50% luminal stenosis were the most important covariates in their final model, we also showed that pre-operative anatomic characteristics including the inflow artery and outflow vein were the most important predictors in our best-performing XGBoost model^10^. Specifically, we demonstrated that higher diameters of the inflow artery and outflow vein were associated with 1-year successful clinical use of the AV access for hemodialysis. Furthermore, we showed that AV fistulae were associated with better 1-year outcomes than AV grafts in terms of clinical use for hemodialysis, potentially due to fewer infectious and/or thrombotic complications, which corroborates previous findings^24^. Overall, the important predictors in our ML model align with clinical intuition and existing literature related to factors associated with successful AV accesses^1^.

Notably, we showed that less than half of the AV accesses created in our cohort were successfully used for hemodialysis at 1-year of follow-up, which corroborates prior literature^6^. This highlights the need for better prediction of planned AV access outcomes to guide pre-operative planning and potentially improve success rates. We demonstrated that several factors were associated with long-term AV access success. We showed that anatomic factors including the inflow artery, outflow vein, access type, and pre-operative ultrasound vein mapping were the most important predictive features, which corroborates previous literature^1^. Our study also demonstrated that younger age and fewer comorbidities, such as dysrhythmias, CHF, and COPD were associated with better AV access outcomes, which aligns with existing knowledge^1^. Within the overall cohort, prescription of cardiovascular risk reduction medications including acetylsalicylic acid and statins was relatively low. Given the high number of vascular comorbidities in this population, there may be an opportunity to improve cardiovascular risk reduction in patients with ESKD^25^. Interestingly, we showed that patients who had prior AV access procedures at any anatomic location were more likely to have successful clinical use of the newly created access for hemodialysis at 1-year of follow-up. This may be because AV accesses are generally created distally first (e.g., radiocephalic) to preserve more proximal access options (e.g., brachiocephalic)^1^. Therefore, when a distal AV access fails and a more proximal AV access is created, the more proximal AV access may have a greater likelihood of success, given the likely larger diameters of the inflow artery/vein and higher flow volumes. This is corroborated by our data and previous work demonstrating that upper arm accesses were more likely to succeed than forearm accesses^26^. For intra-operative variables, we found that the diameters of the inflow artery and outflow vein measured intra-operatively were significantly associated with AV access outcomes. Although pre-operative imaging modalities are improving, the accuracy of vessel diameter measurements may be influenced by the temperature of the room, patient hydration status, recent dialysis, technician skills, tourniquet use, and regional anesthesia, among other factors^27^. However, given that pre-operative inflow artery and outflow vein diameters generally correlate well with intra-operative measurements, these additional variables likely did not contribute significantly to the model^27^. This may explain why intra-operative variables did not improve model performance compared to using only pre-operative variables. For post-operative variables, we found that immediate post-operative complications not requiring access ligation or abandonment, including bleeding, ischemic steal, and thrombosis, were important predictors of long-term AV access success, which corroborates prior literature^28^. Importantly, we demonstrated that pre-operative features can accurately predict 1-year successful clinical use of the index AV access for hemodialysis, while intra-operative variables did not change model performance and post-operative variables marginally improved model performance. This suggests that the ability to accurately predict long-term outcomes following AV access creation can be established pre-procedurally to support decision-making regarding AV access planning, procedural selection, counselling, peri-procedural management, and follow-up^29^.

Our ML models performed better than existing tools for several potential reasons. Compared to traditional logistic regression, advanced ML techniques can better model complex, non-linear relationships between inputs and outputs^30^. This is especially important in health care data, as patient outcomes can be influenced by many demographic, clinical, and system-level factors^31^. Our top-performing algorithm was XGBoost, which has unique advantages including relatively fewer issues with overfitting and faster computing while maintaining precision^32^. Furthermore, XGBoost works well with structured data, which may explain its superior performance compared to more complex algorithms such as neural networks on our dataset^32^.

Importantly, the performance of our models remained robust on subgroup analyses of specific demographic and clinical populations. This is a notable finding given that algorithm bias against underrepresented populations is a frequently encountered issue in ML models^33^. We were likely able to avoid such biases due to the excellent capture of sociodemographic data by VQI^12^. Important to this study, we demonstrated that our models performed well on both AV fistulae and grafts, as well as on different AV access locations, including the forearm, upper arm, lower extremity, or other anatomic location. Therefore, our models are versatile and have clinical utility across a wide range of options available for AV access creation, providing clinicians with a useful tool to support their complex decision-making.

**Fig. 4:**
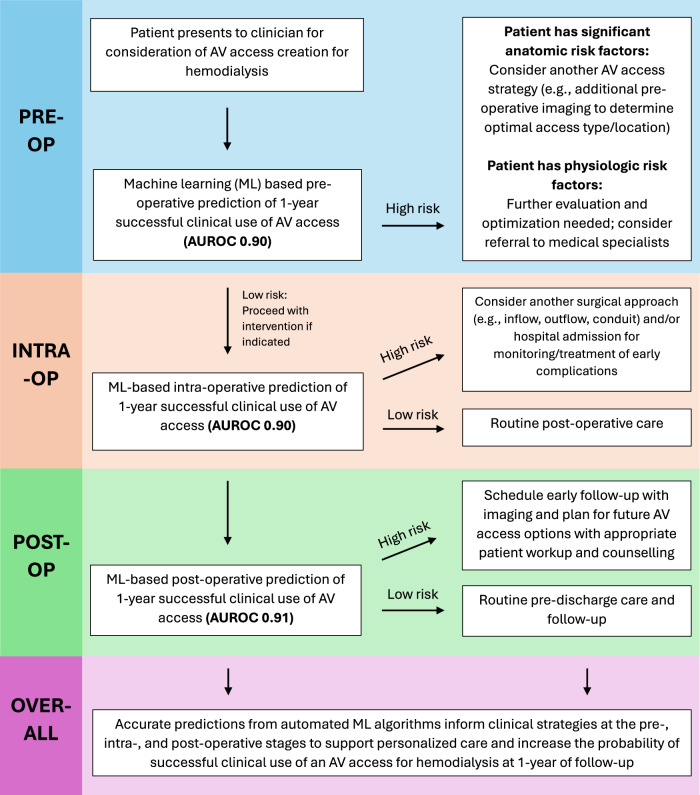
Clinical workflow for the use of machine learning algorithms to guide clinical decision-making at the pre-, intra-, and post-operative stages for patients being considered for arteriovenous access creation. Abbreviations: AUROC (area under the receiver operating characteristic curve), AV (arteriovenous). High risk defined as a model prediction negative for 1-year successful clinical use of an arteriovenous access for hemodialysis. Low risk defined as a model prediction positive for 1-year successful clinical use of an arteriovenous access for hemodialysis.

Our ML models can be used to guide clinical decision-making in several ways for patients being considered for AV access creation. Pre-operatively, a patient predicted to have a low probability (<50%) of 1-year successful clinical use of the AV access for hemodialysis should be further assessed in terms of anatomic and physiologic risk factors^1^. Patients with anatomic risk factors may benefit from further imaging (e.g., ultrasound vein mapping, arterial duplex, arteriogram, or venogram) to support access planning (e.g., decisions on the inflow artery, outflow vein, and/or fistula vs. graft)^1^. Patients with physiologic risk factors, including CHF, COPD, dysrhythmias, or other comorbidities, may benefit from referrals to medical specialists to optimize their cardiovascular risk factors to potentially improve AV access outcomes^1^. Additionally, these patients should receive adequate counselling so that they understand the potential risk of failure and can make informed decisions regarding their care. Intra-operatively, patients predicted to be at high risk for AV access failure (potentially due to intra-operative findings of inadequate artery or vein diameters from vasospasm or other issues) may benefit from choosing a different inflow artery or outflow vein for access creation^1^. Additionally, these patients may benefit from a short admission to hospital for monitoring and treatment of any potential early AV access complications^34^. Post-operatively, patients predicted to have a low probability of long-term AV access success may benefit from close follow-up in clinic with functional imaging^34^. Additionally, for patients at heightened risk for AV access failure, clinicians may begin early planning for future AV access options with appropriate patient workup and counselling. Figure 4 illustrates a proposed clinical workflow for the use of our ML tool in supporting clinical decision-making at the pre-, intra-, and post-operative stages for patients being considered for an AV access creation procedure. Importantly, this workflow highlights the excellent performance of our ML tool in predicting long-term AV access success throughout a patient’s surgical course, which can guide clinical decision-making at each stage of their treatment. These peri-operative decisions guided by our tool have potential to improve patient-centered care and AV access outcomes by supporting individualized patient treatment, monitoring, and counselling. This is particularly important given that the KDOQI ESKD-Life Plan emphasizes the importance of a personalized approach to dialysis access, focused on the right access for the right patient at the right time with continuous monitoring^35^.

The programming code used to develop our ML models is publicly available on GitHub, allowing clinicians involved in the peri-operative management of patients being considered for AV access creation to use our tool. At a system-wide level, our models can be implemented by the over 1000 VQI participating centers^12^. The VQI database managers at these institutions routinely capture the input features used in our ML algorithms^12^. The number of VQI centers has grown considerably from 400 in 2019 to over 1000 in 2023^12^. Recently, the VQI recorded over 1 million procedures^12^. Therefore, our models have broad and growing utility. They also have potential for use beyond VQI sites, as our predictors are commonly captured variables for the routine care of patients being considered for AV access creation^36^. Given the challenges of deploying prediction models into practice, thoughtful consideration of implementation science principles is critical. A key advantage of our ML models is their ability to provide automated risk predictions, thereby enhancing feasibility in busy clinical settings compared to traditional risk predictors that often require manual input of variables^14^. Specifically, our ML algorithms can autonomously extract a patient’s VQI data to generate risk predictions. To facilitate successful implementation of our ML tool, we recommend establishing and supporting data analytics teams at the institutional level. Such teams can provide important benefits to patient care, and their expertise can facilitate the deployment of our ML models^37^.

Our study has several limitations. First, our models were developed with VQI data, a voluntary registry primarily comprising data from North American centers^12^. Model performance may not translate entirely to other healthcare systems, such as single-payer systems outside North America or regions with differing patient demographics, surgical techniques, or post-operative monitoring standards. Future studies are needed to assess the generalizability of model performance beyond VQI sites. Additionally, the VQI-reported endpoint of successful clinical use of an AV access should be validated in future studies. Furthermore, the capture of high-flow related complications may further enhance the clinical utility of AV access outcome prediction models. Second, although we evaluated 6 different ML models, there are other ML models available. We chose these 6 models because of their established efficacy for predicting postoperative outcomes^38^. We achieved excellent performance; however ongoing evaluation of novel ML techniques would be prudent. Third, our cohort included various AV access types (fistulae and grafts) and anatomic locations (forearm, upper arm, lower extremity, or other). This may increase heterogeneity during model training. However, we demonstrated that our models performed well when evaluated on different AV access types and anatomic locations through subgroup analyses. This may improve potential clinical utility as clinicians must consider various access types and locations when planning for an optimal AV access^1^. However, future models trained on a single access type and/or location may provide more granular information. Fourth, most existing models incorporate post-operative ultrasound measurements as input features^10,11^. This information was not available in the VQI database. Although our models achieved excellent performance with pre-operative variables alone, future algorithms that can incorporate both our pre-operative variables and relevant post-operative ultrasound measurements may further enhance overall model performance to guide post-operative clinical decision-making.

In conclusion, we used a large, vascular-specific clinical registry (VQI) to develop robust ML models that can predict 1-year successful clinical use of an AV access with excellent performance using pre-operative data (AUROC’s ≥0.90). Our models can guide clinical decision-making regarding AV access planning, monitoring, and counselling, among other strategies, to facilitate personalized care and potentially improve the probability of AV access success. Importantly, we demonstrated that using pre-operative variables alone in our models can accurately predict long-term AV access outcomes, while intra- and post-operative variables marginally improved model performance. This demonstrates the potential utility of our models for guiding pre-operative decision-making. Notably, our models remained robust across demographic/clinical subpopulations and outperformed existing prediction tools and logistic regression, and therefore, have potential for important utility in the care of patients with kidney failure requiring hemodialysis. Prospective validation of our ML algorithms is planned to confirm clinical utility and impact.

## Methods

### Study approval

The SVS Patient Safety Organization (PSO) Research Advisory Council approved this project and provided the anonymized dataset. Patient consent was not required as the data originated from an anonymized registry.

### Design

This was an ML-based prognostic study with findings reported based on the Transparent Reporting of a Multivariable Prediction Model for Individual Prognosis or Diagnosis + Artificial Intelligence (TRIPOD + AI) statement^39^.

### Dataset

The VQI database is a clinical registry maintained by the SVS PSO with the goal of improving vascular care (www.vqi.org)^12^. Vascular surgeons, interventionalists, and other specialists across more than 1000 academic and community hospitals in the United States, Canada, and Singapore prospectively contribute demographic, clinical, and outcomes data on consecutive eligible vascular patients including information from their index procedure up to approximately 1 year of follow-up^12^. Routine audits comparing submitted data to hospital claims are performed to ensure data accuracy^40^.

### Patient cohort

All patients who underwent surgical AV fistula or graft creation for hemodialysis access from August 1, 2011 to January 2, 2024 in the VQI database were included. The VQI hemodialysis access registry captures AV fistula and graft creation procedures in the upper/lower extremities and other anatomic locations, and excludes dialysis catheter insertions and revisions of an existing access^41^. Patients with no reported access type, inflow artery, or outflow vein were excluded. Patients who underwent endovascular AV fistula creation were also excluded as they accounted for <0.1% of procedures in the hemodialysis access registry.

### Features

Predictor variables (features) used in the ML models were divided into pre-, intra-, and post-operative stages. Given the advantage of ML in handling many input features, all available VQI variables were used to maximize predictive performance. Models were first trained and evaluated using pre-operative features alone, and secondarily trained using intra- and post-operative variables to determine the impact of these features on model performance in a sensitivity analysis. Pre-operative features (*n* = 111) included demographics, comorbidities (including CKD stage and dialysis status), functional status, laboratory investigations (including creatinine and eGFR calculated using the CKD – Epidemiology Collaboration [EPI] 2021 Equation^42^), medications, vascular access history, imaging and anatomic characteristics (including access type [fistula or graft], inflow artery, and outflow vein). To capture the impact of surgeon and center volumes on AV access outcomes, the deidentified physician and center identification numbers were included as pre-operative features.

For sensitivity analysis, intra-operative features (*n* = 22) included the type of anesthesia (local, regional, and/or general), intra-operative target artery and vein diameters, concomitant procedures, and completion imaging studies. Post-operative features (*n* = 17) included immediate post-operative complications (e.g., bleeding, ischemic steal, thrombosis, etc.) not requiring access ligation or abandonment and medical/surgical management of these complications, hospital length of stay, discharge medications, and non-home discharge. A complete list of features and their definitions can be found in Supplementary Tables 1–3.

### Outcomes

The primary outcome was 1-year successful clinical use of the index AV access for hemodialysis, defined as an AV access that can provide the prescribed dialysis consistently with 2 needles for more than two-thirds of dialysis sessions within 4 consecutive weeks, with or without re-interventions, determined through direct clinical follow-up^35^. This outcome is based on follow-up information collected by the participating centers based on dialysis data from recorded treatment sessions and vascular access utilization information from electronic health records^12^. Although VQI primarily includes peri-operative and short-term follow-up data, 1-year dialysis outcomes data is well-captured within the hemodialysis access registry^12^. Therefore, linkage with dialysis databases or other longitudinal follow-up mechanisms beyond VQI was not required. These data are captured and verified by trained VQI database managers at each site, with routine audits comparing submitted data to hospital claims to ensure data accuracy^40^. The primary outcome is equivalent to functional cumulative patency as defined by the National Kidney Foundation KDOQI 2019 Clinical Practice Guidelines for Vascular Access^35^. This primary outcome was chosen because it is clinically relevant, as it requires the AV access to be both anatomically patent and functionally active^35^. A primary/secondary patency endpoint was not chosen because an AV access may be patent but not functional^35^. Similarly, a thrombotic primary endpoint was not chosen as AV accesses are known to suffer from thrombotic or other complications requiring re-interventions but may remain functionally active afterwards^35^. The analysis includes the full period from access creation to maturation, and therefore, accesses that fail to mature are considered to be unsuccessful, which is clinically relevant as they cannot be used for hemodialysis. Secondary outcomes were 1-year AV access thrombosis (defined as complete occlusion of the index AV access by a thrombus) and re-intervention (defined as endovascular or surgical re-intervention on the index AV access to promote maturation and/or to treat a complication)^35^. These diagnostic criteria are based on VQI registry data^12^. Patients who underwent multiple re-interventions were only counted once in this dataset, as re-intervention was considered a binary outcome in our study to simplify predictive modelling. The primary outcome was chosen because accurate prediction of 1-year successful clinical use of an AV access for hemodialysis can provide clinicians with a better understanding of patients who may or may not benefit from a specific AV access creation procedure. This information may guide clinical decision-making regarding the consideration of other AV access approaches, appropriate patient counselling, close post-operative monitoring, and/or other strategies to support AV access maturation and maintenance^43^.

### Model development

We trained 6 different ML models to predict 1-year successful clinical use of an AV access for hemodialysis: XGBoost, random forest, Naïve Bayes classifier, RBF SVM, MLP ANN with a single hidden layer, sigmoid activation function, and cross-entropy loss function, and logistic regression. These models were chosen based on their established efficacy in predicting postoperative outcomes using structured data^38,44,45^. Specifically, these 6 ML models are widely used for surgical outcomes prediction, demonstrating excellent performance on a variety of datasets, procedures, and patient populations^38,44,45^. Logistic regression was selected as the baseline comparator to assess relative model performance because it is the most commonly applied statistical technique in traditional risk prediction tools^46^.

The data was randomly divided into training (70%) and testing (30%) sets. Testing data was reserved for model evaluation and not used for training to ensure fair model assessment. To determine the optimal model hyperparameters, 10-fold cross-validation and grid search were applied to training data^47,48^. Initial analysis demonstrated that the primary outcome occurred in 28,304/59,674 (47.4%) patients in our cohort. To achieve class balance, Random Over-Sample Examples (ROSE) was applied to training data^49^. ROSE uses a smoothed bootstrapping approach to generate new samples from the feature space surrounding the minority class, a commonly used method to support predictive modelling^49^. The models were then evaluated on test set data and ranked based on the primary discriminatory metric of AUROC. The models were compared at the pre-operative stage because predictions at this timepoint offer the most potential to guide clinical decision-making, such as whether to proceed with a specific AV access creation procedure^50^. Our best performing model was XGBoost, which had the following optimized hyperparameters: number of rounds = 200, maximum tree depth = 4, learning rate = 0.05, gamma = 0, column sample by tree = 0.8, minimum child weight = 1, and subsample = 1. Supplementary Table 4 outlines the process for selecting these hyperparameters, as well as the model architecture, feature engineering, and optimized hyperparameters for the 6 ML models.

After identifying the best performing ML model at the pre-operative stage, we performed sensitivity analyses by training additional algorithms using intra- and post-operative data. This approach involved considering different features at each phase of the perioperative course. At the pre-operative stage, only pre-operative characteristics were considered. At the intra-operative stage, both pre- and intra-operative features were used. At the post-operative stage, all pre-, intra-, and post-operative features were inputted into the model. Post-operative variables were included because the immediate post-operative in-hospital course may have an important impact on the long-term success of an AV access^51^. To ensure that post-operative variables did not artificially elevate model performance for predicting 1-year successful clinical use of the AV access for hemodialysis, the only immediate post-operative complications used as input features were those that did not lead to access ligation or abandonment. This multi-phase approach allows clinicians to gain insights into the probability of long-term AV access success/failure at different stages of a patient’s perioperative course, thereby guiding decision-making before, during, and after intervention. This model training method has been previously described, particularly for the development of prediction tools for long-term outcomes^52,53^. Importantly, the focus of this study was on the pre-operative model, and the creation of the additional intra- and post-operative algorithms was primarily to assess the relative impact of intra- and post-operative variables on model performance compared to pre-operative variables alone.

### Statistical analysis

Pre-, intra-, and post-operative features were summarized as means (standard deviation) or medians (interquartile range) for continuous variables and numbers (%) for categorical variables. Differences between patients with and without 1-year successful clinical use of the AV access for hemodialysis were assessed using independent t-tests (continuous variables) or chi-square tests (categorical variables). To account for multiple comparisons, Bonferroni correction was used to set statistical significance. The primary model evaluation metric was AUROC (95% CI), a validated measurement of discriminatory ability that considers both sensitivity and specificity^54^. Differences in AUROC’s between the pre-operative model and the intra- and post-operative models were evaluated using the DeLong test^55^. Secondary performance metrics were accuracy, sensitivity, specificity, PPV, and NPV. To assess model robustness, we plotted calibration curves and calculated Brier scores, a measurement of the agreement between predicted and observed event probabilities^56^. In the final model, feature importance was determined by ranking the top 10 predictors based on variable importance scores (gain), a measurement of the relative importance of individual covariates in contributing to an overall prediction^57^. To assess model bias, we evaluated predictive performance across demographic/clinical subgroups based on age, sex, race, ethnicity, rurality, median ADI percentile, access type (fistula or graft), and access location (forearm, upper arm, lower extremity, or other).

Based on a validated sample size calculator for clinical prediction models, to achieve a minimum AUROC of 0.7 with an outcome rate of approximately 47% and 111 pre-operative features, a minimum sample size of 7730 patients with 3634 events is required^58^. Our cohort of 59,674 patients with 28,304 primary events satisfied this sample size requirement. For variables of interest, missing data was less than 5%; hence, we adopted a complete-case analysis approach, considering only non-missing covariates for each patient. This is a valid analytical method for datasets with minimal missing data (<5%) and reflects predictive modelling of real-world data, which inherently includes missing information^59,60^. Furthermore, data was missing completely at random, with no association between center volume and missing data. Patients who underwent planned two-stage procedures were included once within the cohort, with data from both the primary and secondary procedures accounted for within a single entry. Patients who died, received a kidney transplant, changed dialysis modalities, were pre-dialysis and did not progress to ESKD (defined as CKD stage 5), or were lost to follow-up were censored. In total, there were 1964/59,674 (3.3%) censored cases. Given the relatively low proportion of censored cases, they were considered non-events, with a sensitivity analysis excluding censored observations demonstrating no significant impact on model training or performance. This approach is supported by previous work^61–63^. All analyses were conducted using R version 4.3.1^64^.

## Supplementary information

Supplementary information

## Data Availability

The data used for this study comes from the Vascular Quality Initiative Database, which is maintained by the Society for Vascular Surgery Patient Safety Organization. Access and use of the data requires approval through an application process available at https://www.vqi.org/data-analysis/.

## Code availability

The complete code used for model development and evaluation in this project is publicly available on GitHub: https://github.com/benli12345/AV-ML-VQI.
