# Supplementary material for: Predicting 1-year successful clinical use of an arteriovenous access for hemodialysis using machine learning
Source: NPJ Digit Med. 2025 Dec 4;9:15. doi: 10.1038/s41746-025-02187-9 (PMC12779959; doi:10.1038/s41746-025-02187-9)
Supplement: Supplementary file 1 — Supplementary information [file 41746_2025_2187_MOESM1_ESM.pdf]

Supplementary Table 1. Pre-operative features for machine learning models

Features (n = 111)	Definition based on Vascular Quality Initiative Data Dictionary
**Logistics**	
Region	Region of procedure (deidentified)
Center	Center of procedure (deidentified)
Physician	Physician who performed procedure (deidentified)
Intervention year	Year of intervention
Intervention month	Month of intervention
Intervention weekday	Day of week that intervention was performed
**Demographics**	
Age	Age in years
Sex	Male or female
Body mass index	Weight in kg / height in m^2
Race	Patient-reported race including White, Black, Asian, American Indian or Alaskan Native, Native Hawaiian or other Pacific Islander, more than 1 race, or unknown/other
Ethnicity	Hispanic or non-Hispanic
Primary insurer	Primary method of health insurance including Medicare, Medicaid, Commercial, Medicare Advantage, Military or Veterans Affairs, Non-US Insurance, self-pay (uninsured), or unknown/other
Rural residence	Defined based on the patient's primary rural-urban commuting area (RUCA) code based on the most recent publicly available dataset. Rural residence is RUCA code 10 (https://www.ers.usda.gov/data-products/rural-urban-commuting-area-codes/documentation//).
Median Area Deprivation Index (ADI)	National percentile rank based on the most recent publicly available dataset. Calculated by taking the median ADI national percentile rank among all 9-digit zip code records that contain the patient's 5-digit zip code prefix (https://www.neighborhoodatlas.medicine.wisc.edu/). A higher number indicates a greater level of socioeconomic disadvantage, accounting for factors such as income, education, employment, and housing quality.
Performance site	Setting where intervention was performed: 1) hospital outpatient, 2) hospital inpatient, 3) ambulatory center, 4) office, 5) not reported
Transfer status	Transferred from another hospital or rehabilitation unit to center of intervention
**Comorbidities**	
Smoking status	Current, prior (quit ≥ 1 month ago), or never

Features (n = 111)	Definition based on Vascular Quality Initiative Data Dictionary
Hypertension	Documented in history or recorded blood pressure > 130/80 on 3 or more occasions
Diabetes	Documented in history or receiving anti-hyperglycemic medications including insulin
Coronary artery disease	History of myocardial infarction, stable angina, or unstable angina
Coronary artery bypass graft	Prior coronary artery bypass graft
Percutaneous coronary intervention	Prior percutaneous coronary intervention
Dysrhythmia	Dysrhythmia at the time of treatment as documented in the medical record or electrocardiogram, including atrial fibrillation/flutter, supraventricular arrhythmia, ventricular dysrhythmia, atrioventricular block, pacemaker, implantable cardioverter defibrillator, or other dysrhythmia
Congestive heart failure	Documented in history and severity classified based on the New York Heart Association (NYHA) heart failure classification
Chronic obstructive pulmonary disease	Documented in history and whether patient is not treated, on medications, or home oxygen
Peripheral artery disease	Documented peripheral artery disease including symptom status: asymptomatic, claudication, rest pain, or tissue loss
Intravenous drug use	Past history (> 12 months) or current history of intravenous recreational drug use within the past 12 months
HIV status	1) No history of HIV, 2) Positive HIV test, 3) Positive HIV test with current infection, 4) Not available or not reported
Chronic kidney disease stage	Stage 1: eGFR is at 90 or above, stage 2: eGFR 60-89, stage 3: eGFR 30-59, stage 4: eGFR 15-30, stage 5 eGFR < 15
Dialysis status	1) Functioning transplant, never on dialysis, 2) functioning transplant, previously on dialysis, 3) on hemodialysis, 4) pre-dialysis (patient anticipated to require dialysis in the future for treatment of kidney failure, but is not currently undergoing dialysis), 5) on peritoneal dialysis
American Society of Anesthesiologists classification	1 (normal/healthy), 2 (mild systemic disease), 3 (severe systemic disease), 4 (severe systemic disease that is a constant threat to life), 5 (moribund, not expected so survive without operation)
**Functional status**	
Living status	Last living status prior to hospitalization (home, nursing home, or homeless)
Ambulatory status	1) Ambulatory independently, 2) ambulatory with assistance, 3) wheelchair-dependent, 4) bedridden

Features (n = 111)	Definition based on Vascular Quality Initiative Data Dictionary
**Laboratory investigations**	
Hemoglobin	Most recent pre-operative hemoglobin within 6 months prior to surgery (g/L)
Creatinine	Most recent pre-operative creatinine within 6 months prior to surgery (umol/L)
eGFR	Calculated using CKD-EPI Creatinine Equation (https://www.kidney.org/professionals/gfr_calculator)
**Medications**	Taken within 36 hours of procedure
Acetylsalicylic acid	Includes drugs that contain acetylsalicylic acid
P2Y12 antagonist	Includes clopidogrel, prasugrel, ticlopidine, and ticagrelor
Statin	Includes atorvastatin, fluvastatin, lovastatin, pitavastatin, pravastatin, rosuvastatin, simvastatin, or a combination medication including a statin
Anticoagulant	Includes warfarin, dabigatran, rivaroxaban, or other anticoagulant
**Vascular access history**	
Current access	1) Percutaneous catheter, 2) tunnelled catheter, 3) prior AV access
Previous AV access	History of previous surgical or endovascular AV fistula or graft
Number of previous AV accesses	Number of previous surgical or endovascular AV fistulae or grafts
Previous AV fistulae	Number of previous AV fistula creation procedures at any location
Previous AV grafts	Number of previous AV graft creation procedures at any location
Previous forearm fistula	Previous forearm fistula below the elbow (right, left, or bilateral)
Previous forearm graft	Previous forearm graft below the elbow (right, left, or bilateral)
Previous upper arm fistula	Previous upper arm fistula at or above the elbow (right, left, or bilateral)
Previous upper arm graft	Previous upper arm graft at or above the elbow (right, left, or bilateral)
Previous tunnelled catheter	Previous semi-permanent catheter tunnelled under the skin (right, left, or bilateral)
Previous lower extremity AV access	Previous AV access in the lower extremity (right, left, or bilateral)
Previous other AV access	Previous AV access not covered above, such as direct aortic access or axillary-subclavian access
Side of most recent AV access procedure if applicable	Right or left
Side of 2nd most recent AV access procedure if applicable	Right or left

**Features (n = 111)**	**Definition based on Vascular Quality Initiative Data Dictionary**
Side of 3rd most recent AV access procedure if applicable	Right or left
Side of 4th most recent AV access procedure if applicable	Right or left
Type of most recent AV access procedure if applicable	1) Surgical AV fistula, 2) AV graft, 3) endovascular AV fistula
Type of 2nd most recent AV access procedure if applicable	1) Surgical AV fistula, 2) AV graft, 3) endovascular AV fistula
Type of 3rd most recent AV access procedure if applicable	1) Surgical AV fistula, 2) AV graft, 3) endovascular AV fistula
Type of 4th most recent AV access procedure if applicable	1) Surgical AV fistula, 2) AV graft, 3) endovascular AV fistula
Location of most recent AV access procedure if applicable	1) Forearm 2) upper arm, 3) lower extremity, 4) other
Location of 2nd most recent AV access procedure if applicable	1) Forearm 2) upper arm, 3) lower extremity, 4) other
Location of 3rd most recent AV access procedure if applicable	1) Forearm 2) upper arm, 3) lower extremity, 4) other
Location of 4th most recent AV access procedure if applicable	1) Forearm 2) upper arm, 3) lower extremity, 4) other
Previous or current central venous catheter for dialysis	Presence or absence of previous or current central venous catheter for dialysis
Previous right-sided central venous catheter for dialysis	Presence or absence of previous right-sided central venous catheter for dialysis
Previous left-sided central venous catheter for dialysis	Presence or absence of previous left-sided central venous catheter for dialysis

**Features (n = 111)**	**Definition based on Vascular Quality Initiative Data Dictionary**
Current right-sided central venous catheter for dialysis	Presence or absence of current right-sided central venous catheter for dialysis
Current left-sided central venous catheter for dialysis	Presence or absence of current left-sided central venous catheter for dialysis
Previous or current central venous catheter or device not for dialysis	Presence or absence of previous or current central venous catheter or device not for dialysis
Previous right-sided central venous catheter or device not for dialysis	Presence or absence of previous right-sided central venous catheter or device not for dialysis
Previous left-sided central venous catheter or device not for dialysis	Presence or absence of previous left-sided central venous catheter or device not for dialysis
Current right-sided central venous catheter or device not for dialysis	Presence or absence of current right-sided central venous catheter or device not for dialysis
Current left-sided central venous catheter or device not for dialysis	Presence or absence of current left-sided central venous catheter or device not for dialysis
Previous right-sided PICC line	Presence or absence of previous right-sided PICC line
Previous right-sided subcutaneous venous access port	Presence or absence of previous right-sided subcutaneous venous access port (e.g., port-a-cath)
Previous right-sided cardiac rhythm device	Presence or absence of previous right-sided cardiac rhythm device
Current right-sided PICC line	Presence or absence of current right-sided PICC line
Current right-sided subcutaneous venous access port	Presence or absence of current right-sided subcutaneous venous access port (e.g., port-a-cath)
Current right-sided cardiac rhythm device	Presence or absence of current right-sided cardiac rhythm device
Previous left-sided PICC line	Presence or absence of previous left-sided PICC line
Previous left-sided subcutaneous venous access port	Presence or absence of previous left-sided subcutaneous venous access port (e.g., port-a-cath)

**Features (n = 111)**	**Definition based on Vascular Quality Initiative Data Dictionary**
Previous left-sided cardiac rhythm device	Presence or absence of previous left-sided cardiac rhythm device
Current left-sided PICC line	Presence or absence of current left-sided PICC line
Current left-sided subcutaneous venous access port	Presence or absence of current left-sided subcutaneous venous access port (e.g., port-a-cath)
Current left-sided cardiac rhythm device	Presence or absence of current left-sided cardiac rhythm device
Previous or current lower extremity tunnelled catheter	Presence or absence of previous or current lower extremity tunnelled catheter
Previous right-sided lower extremity tunnelled catheter	Presence or absence of previous right-sided lower extremity tunnelled catheter
Previous left-sided lower extremity tunnelled catheter	Presence or absence of previous left-sided lower extremity tunnelled catheter
Current right-sided lower extremity tunnelled catheter	Presence or absence of current right-sided lower extremity tunnelled catheter
Current left-sided lower extremity tunnelled catheter	Presence or absence of current left-sided lower extremity tunnelled catheter
Other previous vascular access	Presence or absence of other vascular access not covered above
Previous transhepatic catheter	Presence or absence of previous transhepatic catheter
Previous translumbar catheter	Presence or absence of previous translumbar catheter
Previous axillary-axillary graft	Presence or absence of previous axillary-axillary graft
Previous other vascular access	Presence or absence of previous other vascular access not covered above
**Imaging and anatomic characteristics**	
Pre-operative imaging	Any pre-operative imaging done for vascular access planning
Pre-operative arterial duplex	Pre-operative arterial duplex performed for vascular access planning
Pre-operative arteriogram	Pre-operative arteriogram performed for vascular access planning

**Features (n = 111)**	**Definition based on Vascular Quality Initiative Data Dictionary**
Pre-operative ultrasound vein mapping	Pre-operative ultrasound vein mapping performed for vascular access planning
Pre-operative venogram	Pre-operative venogram performed for vascular access planning
Pre-operative artery diameter	Pre-operative diameter of target artery in the area of the anastomosis determined on imaging
Pre-operative vein diameter	Pre-operative diameter of target vein in the area of the anastomosis determined on imaging
Side of procedure	Body side where AV access was created
Access type	AV fistula or AV graft (endovascular AV fistula excluded)
Inflow artery	Anatomic location of arterial anastomosis: 1) Radial, snuffbox, 2) Radial, wrist, 3) Radial, forearm, 4) Radial, mid-forearm, 5) Radial, antecubital, 6) Radial, high takeoff, 7) Ulnar, wrist, 8) Ulnar, mid-forearm, 9) Ulnar, antecubital, 10) Brachial, antecubital, 11) Brachial, upper arm, 12) Axillary, 13) Common femoral artery, 14) Superficial femoral artery, 15) Other
Outflow vein	Anatomic location of intended outflow vein: 1) Cephalic, forearm, 2) Cephalic, upper arm, 3) Basilic, forearm, 4) Basilic, upper arm, 5) Brachial, upper arm, 6) Axillary, 7) Saphenous, 8) Femoral, 9) Other
Planned 2 stage procedure	Planned 2 stage procedure intended to make access more easily punctured
Basilic vein transposition	Anastomosis of upper arm basilic vein to brachial artery and relocation of the vein to a more superficial location (right, left, or bilateral)
Pre-operative endovascular arterial intervention	Pre-operative balloon angioplasty or stent to treat a stenosis of the planned access artery or an artery more proximal to this point
Pre-operative endovascular venous intervention	Pre-operative balloon angioplasty or stenting of the target vein or a more proximal/distal segment

Abbreviations: HIV (human immunodeficiency virus), eGFR (estimated glomerular filtration rate), AV (arteriovenous), PICC (peripherally inserted central catheter), CKD EPI (Chronic Kidney Disease Epidemiology Collaboration).

Supplementary Table 2. Intra-operative features for machine learning models

Features (n = 22)	Definition based on Vascular Quality Initiative Data Dictionary
Anesthesia	1. Local anesthesia (sedation alone),<br>2. Regional anesthesia (epidural or spinal), or<br>3. General anesthesia (mask airway, laryngeal mask airway, or intubation)
Peri-operative antibiotics	First or second-generation cephalosporins were ordered just before the operation
Antibiotics given within 1 hour of intervention	Antibiotics given within 1 hour prior to skin incision or vascular access
Antibiotics stopped within 24 hours of intervention	Antibiotics stopped within 24 hours of intervention end time
Intra-operative target artery diameter	Target artery diameter measured at the time of the operation based on outer diameter at initial exposure
Intra-operative target vein diameter	Target vein diameter measured at the time of the operation based on outer diameter at initial exposure
Concomitant procedures	
No concomitant procedures	Absence of any concomitant procedure
Venous angioplasty	Angioplasty of target vein
Venous stent	Stenting of target vein
Arterial angioplasty	Angioplasty of target artery
Arterial stent	Stenting of target artery
Arterial endarterectomy	Endarterectomy of target artery
Venous branch ligation	Venous branch ligation of target vein
Surgical access patch angioplasty	Patch angioplasty of target artery and/or vein
Superficialization	Superficialization of target vein
Lipectomy	Lipectomy to support AV access creation and/or superficialization
Liposuction	Liposuction to support AV access creation and/or superficialization
Other	Other concomitant procedures not covered above
Completion study	
No completion study	Absence of any completion study
Completion doppler	Doppler used to assess success of AV access creation
Completion duplex ultrasound	Duplex ultrasound used to assess success of AV access creation
Completion fistulogram	Fistulogram used to assess success of AV access creation

Abbreviation: AV (arteriovenous).

Supplementary Table 3. Post-operative features for machine learning models

**Features (n = 17)**	**Definition based on Vascular Quality Initiative Data Dictionary**
Immediate post-operative complications not requiring access ligation or abandonment	
Bleeding	AV access bleeding not requiring ligation
Ischemic steal	AV access ischemic steal not requiring ligation
Access thrombosis	AV access thrombosis not requiring abandonment
Other complication requiring re-intervention	Other AV access complication requiring re-intervention
Bleeding management	1) Resolved spontaneously, 2) resolved with medical treatment to correct coagulation, 3) requiring surgical or endovascular re-intervention
Ischemic steal management	1) Observation, 2) banding, 3) proximalization, 4) revascularization using distal inflow, 5) distal revascularization and interval ligation, 6) distal revascularization
Thrombosis management	
Open surgical thrombectomy	Open surgical thrombectomy to treat AV access thrombosis
Endovascular mechanical thrombectomy	Endovascular mechanical thrombectomy to treat AV access thrombosis
Thrombolysis	Thrombolysis to treat AV access thrombosis
Revision	Surgical revision to treat AV access thrombosis
Total hospital length of stay	Time from admission to discharge (days)
Discharge medications	Prescribed at discharge
Any change in medications	Any change between pre-operative and discharge medications
Acetylsalicylic acid	Includes drugs that contain acetylsalicylic acid
P2Y12 antagonist	Includes clopidogrel, prasugrel, ticlopidine, and ticagrelor
Statin	Includes atorvastatin, fluvastatin, lovastatin, pitavastatin, pravastatin, rosuvastatin, simvastatin, or a combination medication including a statin
Anticoagulant	Includes warfarin, dabigatran, rivaroxaban, or other anticoagulant
Non-home discharge	Discharge to nursing home, rehabilitation unit, or other hospital

Abbreviation: AV (arteriovenous).

Supplementary Table 4. Selection of model hyperparameters using grid search and cross validation

Hyperparameter	Values tested through grid search and cross validation*	Optimal weights chosen to maximize AUROC
**Extreme Gradient Boosting (XGBoost)**		
Number of rounds	50, 100, 150, 200, 250, 300, 350, 400, 450, 500	200
Maximum tree depth	2, 3, 4, 5, 6, 7, 8, 9	4
Learning rate	0.4, 0.3, 0.2, 0.1, 0.05, 0.01, 0.001	0.05
Gamma	0, 0.1, 1, 1.5, 2	0
Column sample by tree	0.5, 0.6, 0.7, 0.8, 0.9, 1	0.8
Minimum child weight	1, 3, 5, 7, 10	1
Subsample	0.5, 0.6, 0.7, 0.8, 0.9, 1	1
**Random forest**		
Number of randomly drawn candidate variables (mtry)	2, 3, 4	3
Minimum node size	10, 20	20
**Naïve bayes classifier**		
Kernel	True, false	True
Laplace correction	0, 1, 2, 3, 4, 5	1
Bandwidth adjustment	0, 1, 2, 3, 4, 5	3
**Support vector machine**		
Regularization parameter (C-value)	0.001, 0.01, 0.1, 1, 10, 100, 10000	0.1
**Artificial neural network**		
Number of units in hidden layer	10, 20, 30, 40, 50	30
Weight decay	0.1, 0.2, 0.3, 0.4, 0.5, 0.6, 0.7, 0.8, 0.9, 1	0.8
**Logistic regression**		
Regularization parameter (C-value)	0.001, 0.01, 0.1, 1, 10, 100, 10000	0.1

*Grid search and cross validation are exhaustive methods that iteratively train and evaluate models using every combination of specified hyperparameter values and selects the set of hyperparameter values that optimize model performance.

Abbreviation: AUROC (area under the receiver operating characteristic curve).

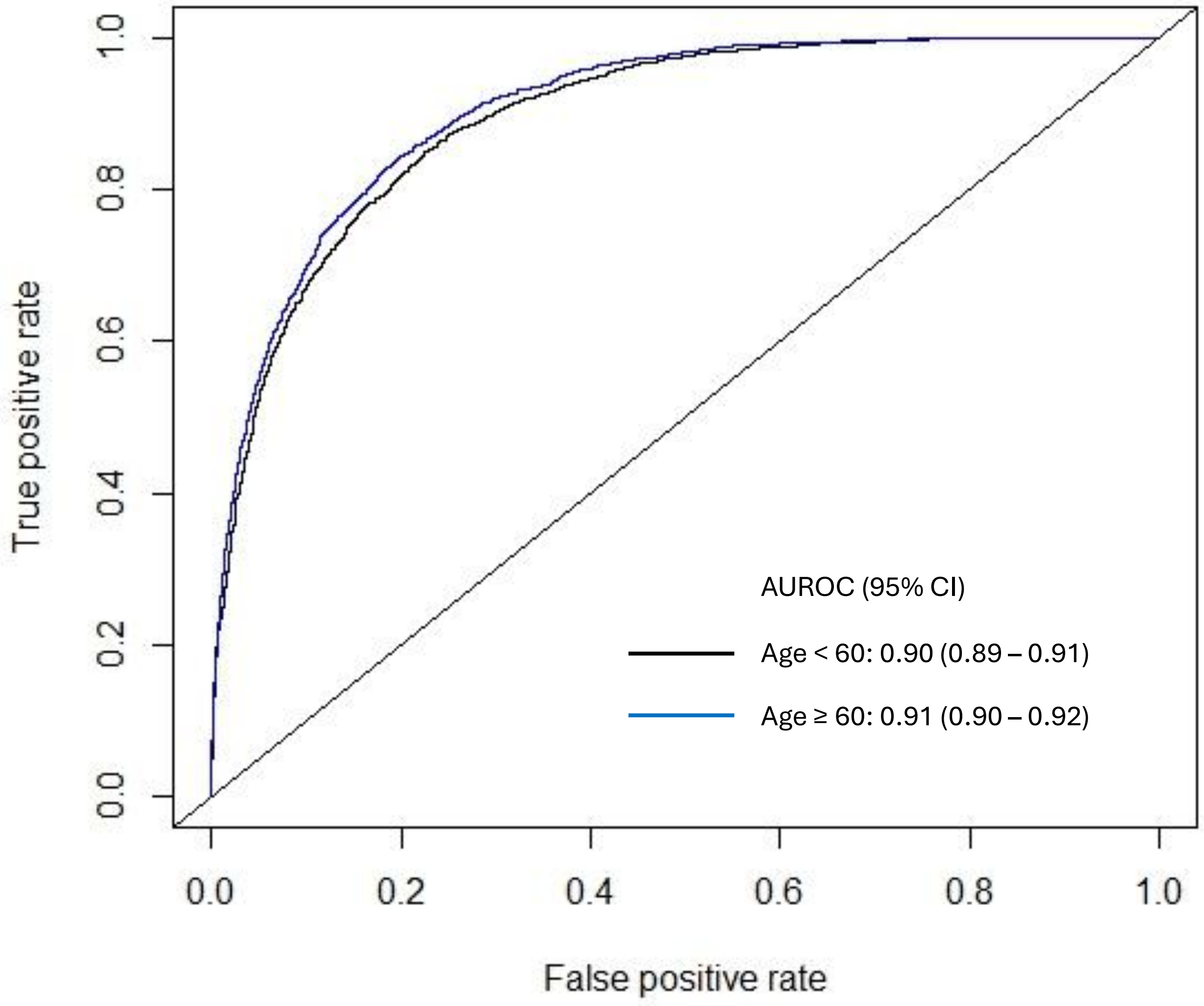

Supplementary Figure 1. Receiver operating characteristic curve for 1-year successful clinical use of an arteriovenous access for hemodialysis using Extreme Gradient Boosting (XGBoost) model at the pre-operative stage with subgroup analysis based on age. AUROC (area under the receiver operating characteristic curve), CI (confidence interval).

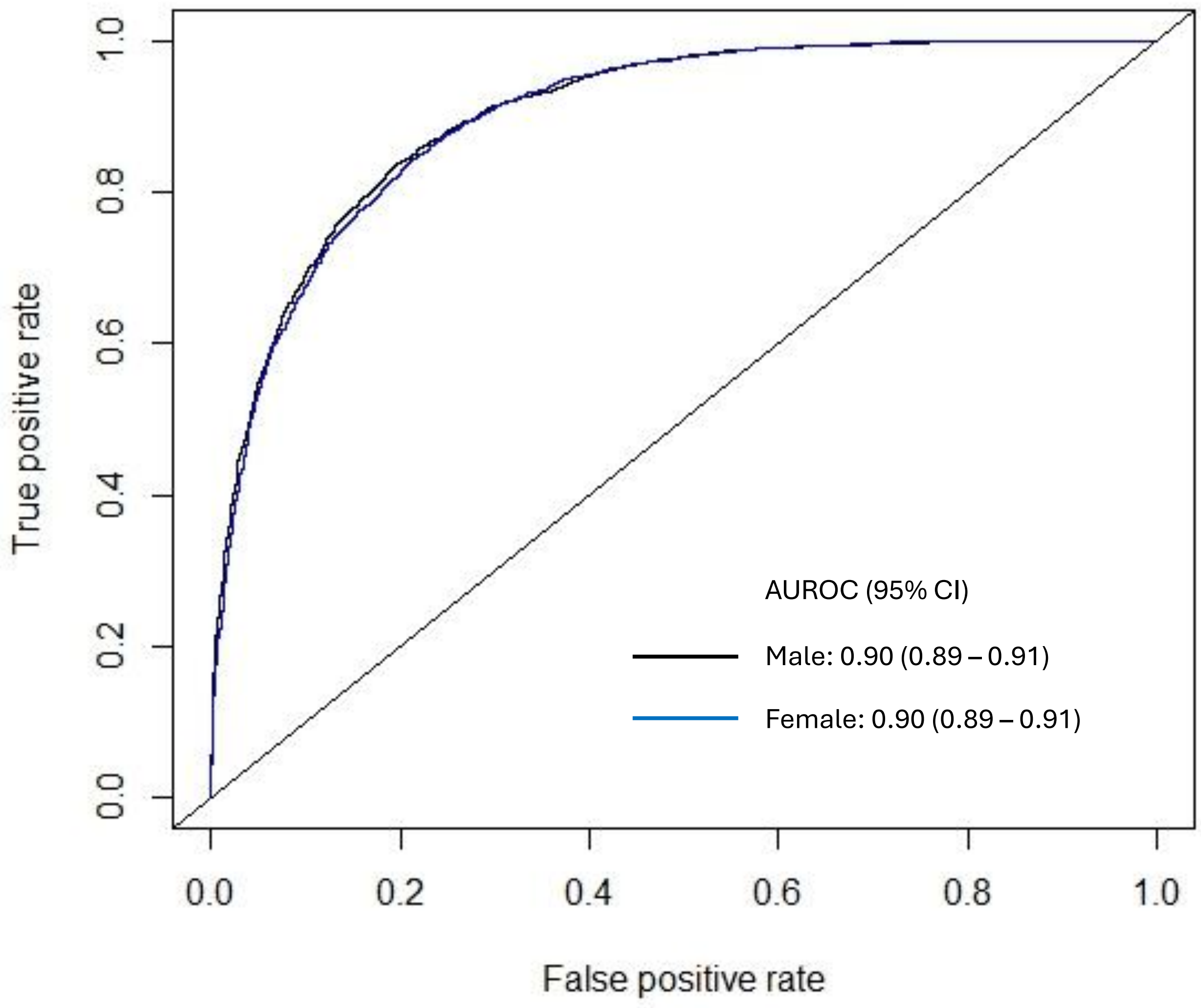

Supplementary Figure 2. Receiver operating characteristic curve for 1-year successful clinical use of an arteriovenous access for hemodialysis using Extreme Gradient Boosting (XGBoost) model at the pre-operative stage with subgroup analysis based on sex. AUROC (area under the receiver operating characteristic curve), CI (confidence interval).

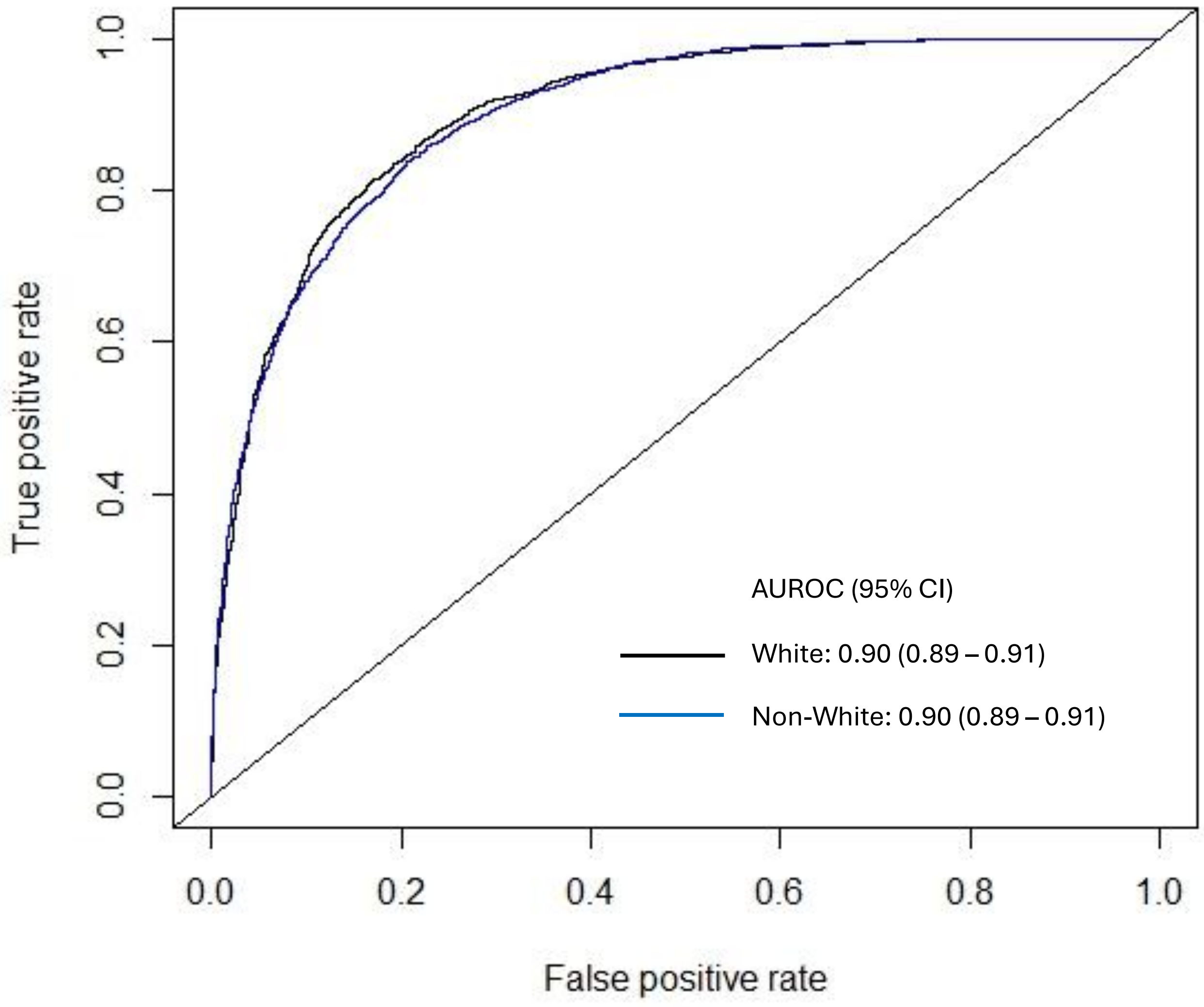

Supplementary Figure 3. Receiver operating characteristic curve for 1-year successful clinical use of an arteriovenous access for hemodialysis using Extreme Gradient Boosting (XGBoost) model at the pre-operative stage with subgroup analysis based on race. AUROC (area under the receiver operating characteristic curve), CI (confidence interval).

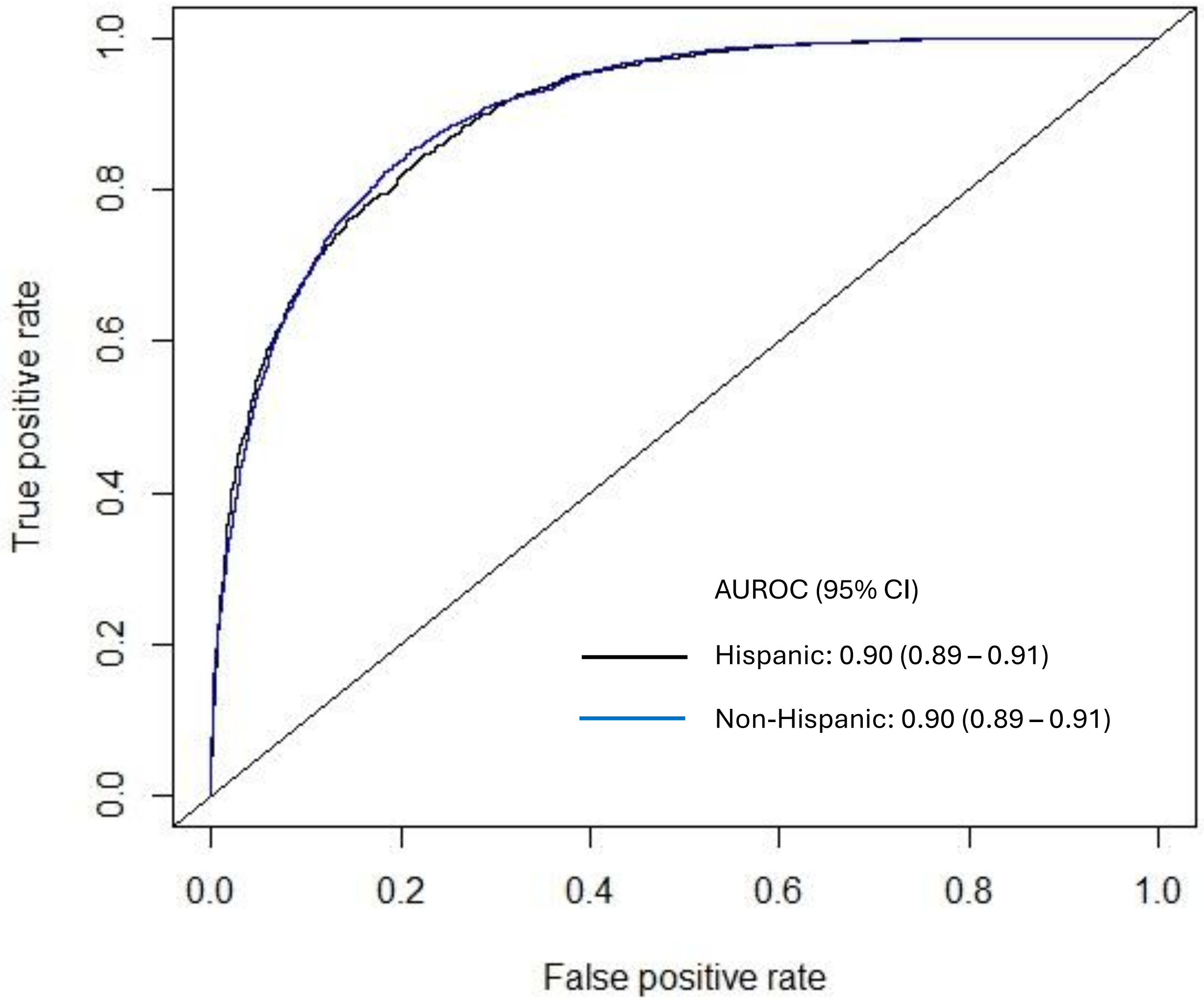

Supplementary Figure 4. Receiver operating characteristic curve for 1-year successful clinical use of an arteriovenous access for hemodialysis using Extreme Gradient Boosting (XGBoost) model at the pre-operative stage with subgroup analysis based on ethnicity. AUROC (area under the receiver operating characteristic curve), CI (confidence interval).

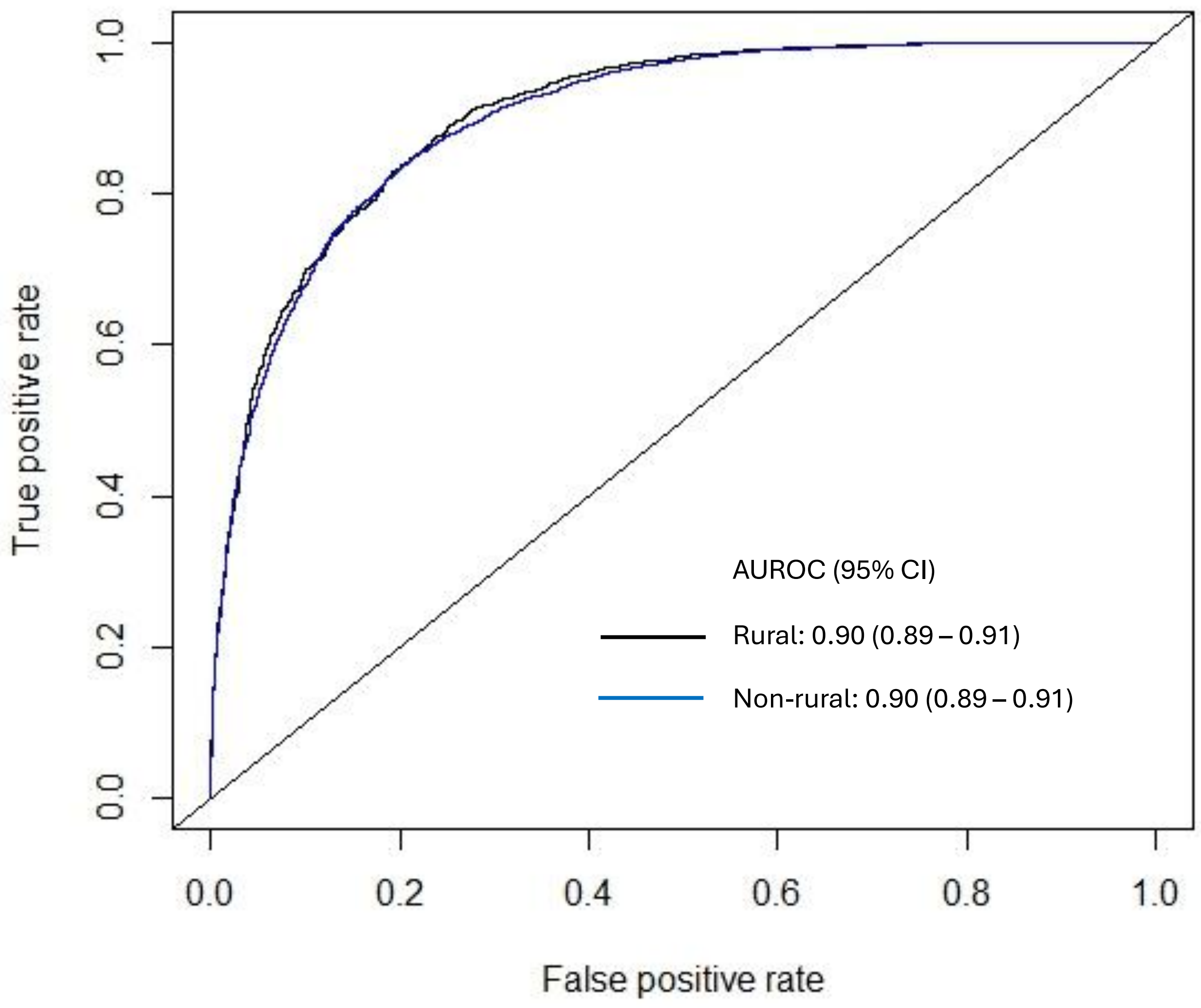

Supplementary Figure 5. Receiver operating characteristic curve for 1-year successful clinical use of an arteriovenous access for hemodialysis using Extreme Gradient Boosting (XGBoost) model at the pre-operative stage with subgroup analysis based on rurality of residence. AUROC (area under the receiver operating characteristic curve), CI (confidence interval).

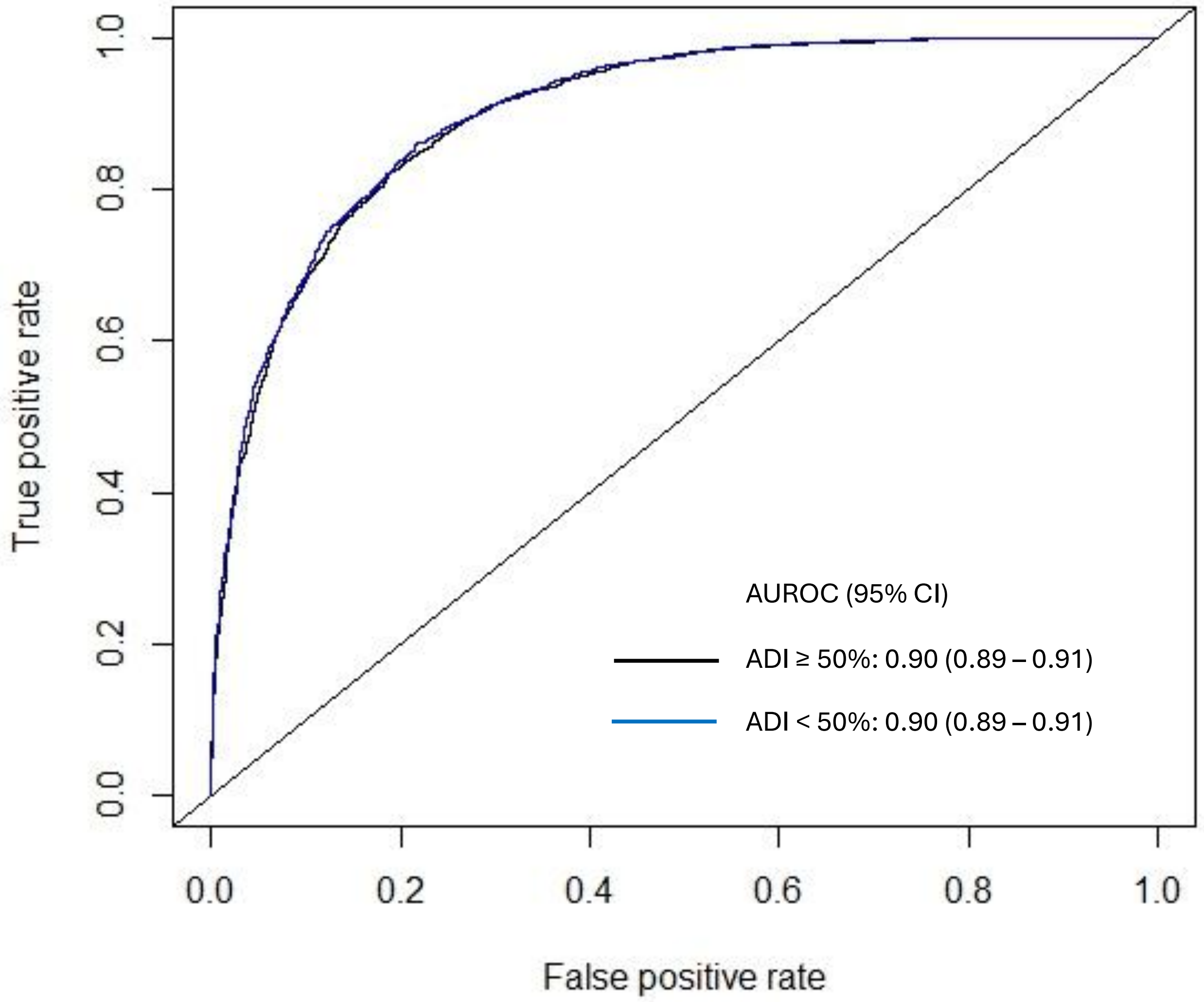

Supplementary Figure 6. Receiver operating characteristic curve for 1-year successful clinical use of an arteriovenous access for hemodialysis using Extreme Gradient Boosting (XGBoost) model at the pre-operative stage with subgroup analysis based on median Area Deprivation Index (ADI) percentile. AUROC (area under the receiver operating characteristic curve), CI (confidence interval).

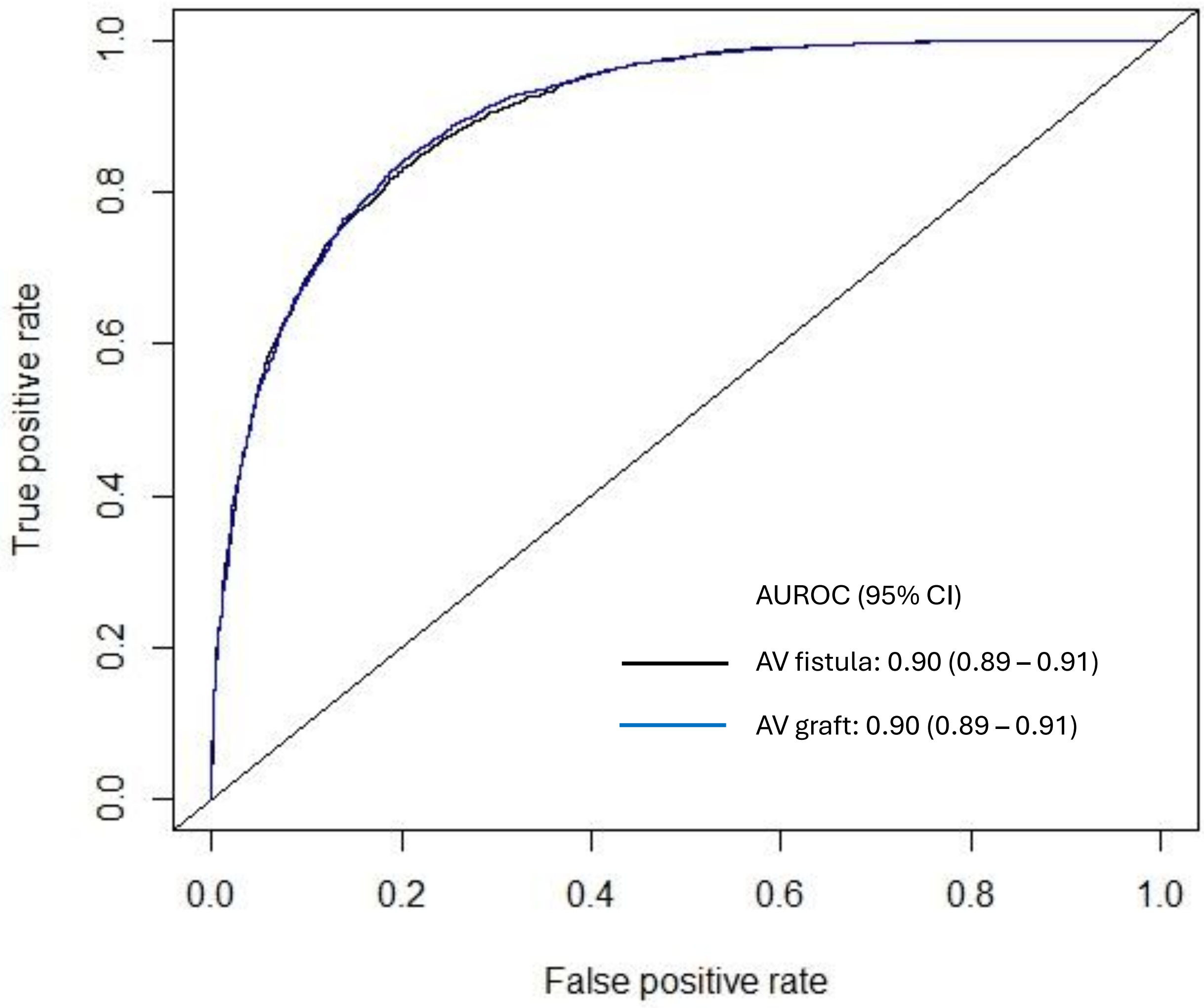

Supplementary Figure 7. Receiver operating characteristic curve for 1-year successful clinical use of an arteriovenous access for hemodialysis using Extreme Gradient Boosting (XGBoost) model at the pre-operative stage with subgroup analysis based on access type. AUROC (area under the receiver operating characteristic curve), CI (confidence interval), AV (arteriovenous).

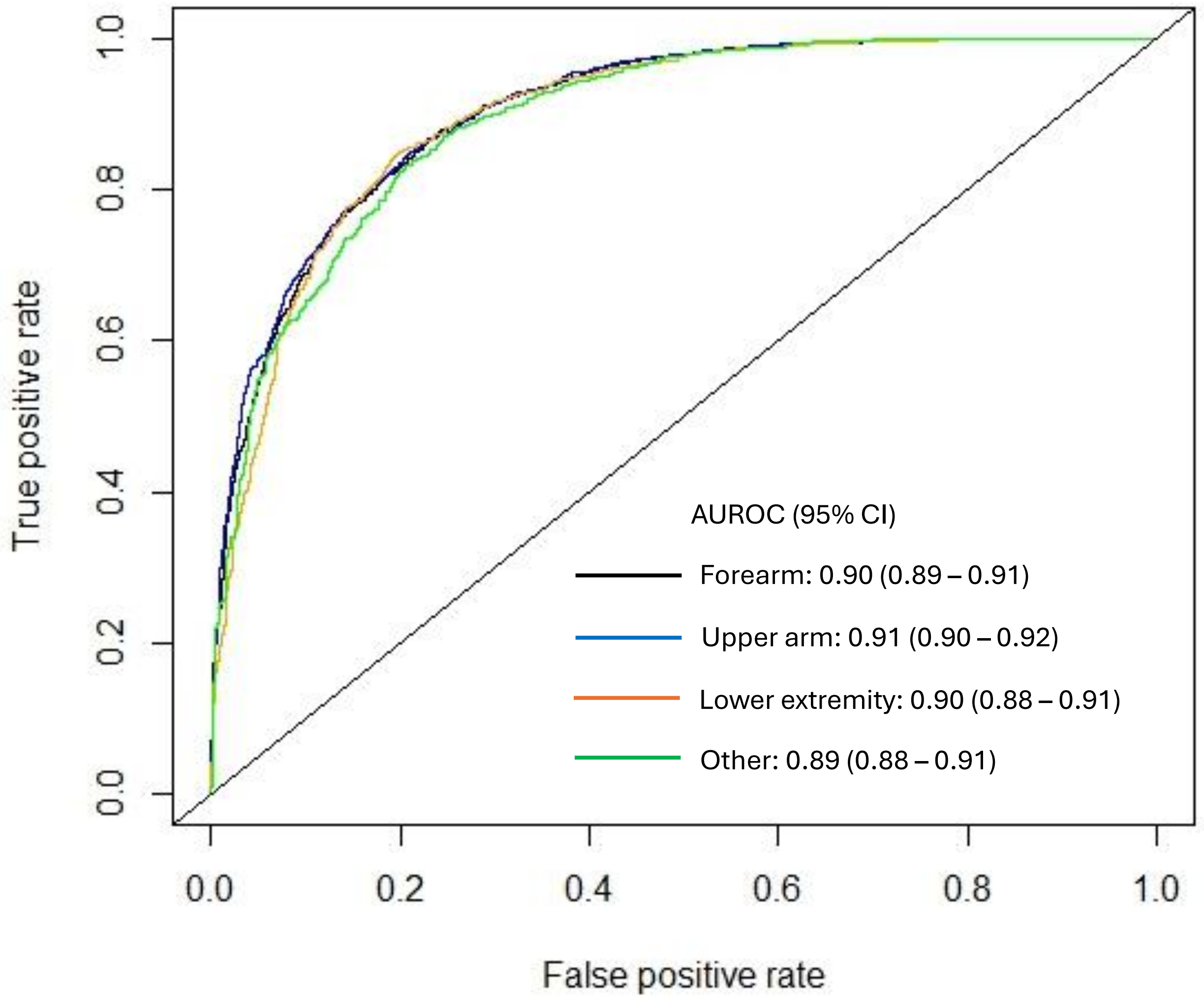

Supplementary Figure 8. Receiver operating characteristic curve for 1-year successful clinical use of an arteriovenous access for hemodialysis using Extreme Gradient Boosting (XGBoost) model at the pre-operative stage with subgroup analysis based on access location. AUROC (area under the receiver operating characteristic curve), CI (confidence interval), AV (arteriovenous).
